# Interpretable Bearing Fault Diagnosis Using Synchronized Acoustic and Rotational-Speed Sensing Under Variable-Speed Conditions

**DOI:** 10.3390/s26206581

**Published:** 2026-10-16

**Authors:** Na Dong, Yu Feng, Xiaodong Liu, Kuan Shi, Qingjun Hu

**Affiliations:** 1 The International Joint Institute of Tianjin University, Fuzhou, Tianjin University, Tianjin 300072, China; 2 Tianjin Research Institute of Electric Science Co., Ltd., Tianjin 300180, China

**Keywords:** rolling bearing, acoustic sensing, variable-speed condition monitoring, order-synchronous analysis, interpretable fault diagnosis, Kolmogorov–Arnold network

## Abstract

**Highlights:**

**What are the main findings?**

- A synchronized acoustic–speed framework establishes an order-consistent representation for bearing diagnosis under continuous varying-speed conditions.
- OSAP-KANFormer combines physics-constrained amplitude–phase learning with concept-based KAN classification to provide explicit class-evidence pathways.

**What are the implications of the main findings?**

- The proposed approach reduces the dependence of acoustic diagnosis on speed-specific feature distributions in practical variable-speed monitoring scenarios.
- The concept-mediated decision pathway offers a more transparent alternative to conventional black-box fault classifiers.

**Abstract:**

Variable-speed operation causes fixed-duration acoustic frames to cover different shaft angles, reducing the mechanical consistency of bearing-fault features. This study proposes OSAP-KANFormer, an interpretable diagnostic framework using synchronized acoustic and rotational-speed measurements. Equal-angle resampling establishes a common mechanical scale, while a Hilbert-initialized learnable analytic layer and reciprocal cross-attention model amplitude–phase information. The final decision passes through predicted physical concepts and a sparse additive Kolmogorov–Arnold classifier. Under source-record-separated evaluation, the method achieved a Macro-F1 of 97.3% on a fixed-speed benchmark and 92.4% in leave-one-speed-out evaluation for previously observed bearing entities. Compared with ASFE-Transformer, Macro-F1 increased by 7.3 percentage points under unseen speeds and by 9.2 percentage points in constant-speed-to-variable-speed transfer. A separately trained external-dataset evaluation achieved 87.0% under a compound speed–load–microphone shift. Eleven physical concepts obtained a mean prediction R^2^ of 0.891. The results support the contribution of mechanical-angle alignment to variable-speed diagnosis and provide an explicit concept-level decision pathway. Applicability remains dependent on speed-reference quality, concept coverage, and the evaluated transfer setting.

## 1. Introduction

Rolling bearings are safety-critical components in motors, gearboxes, pumps, and production equipment, and local defects may develop into performance degradation, secondary damage, and unplanned downtime [1,2]. Vibration remains the dominant monitoring modality because of its sensitivity to structural response, whereas acoustic monitoring offers flexible installation and minimal interference with machine operation [3,4]. Acoustic methods are particularly useful when conventional accelerometers are constrained by installation space, temperature, or cabling. However, the acoustic field is not a direct replica of bearing vibration; it is shaped by rolling and sliding interactions, structural transmission paths, airborne radiation, reflections, and other mechanical background sources. Consequently, diagnostically relevant acoustic transients are typically short, strongly non-stationary, and dependent on operating conditions. Deep convolutional networks can learn local acoustic patterns directly from raw waveforms, while attention-based models improve the representation of long-range relationships among transient events [5,6,7]. Lightweight convolutional Transformers, including LiConvFormer and LSFConvformer, provide a useful balance between global modeling and deployment efficiency [8,9]. The recently proposed ASFE-Transformer established a coherent acoustic architecture by constructing quadrature channels with a Hilbert analytic representation, forming phoneme-like tokens from short overlapping frames, and combining global attention with local token-axis convolution through an SD-GLU module [10]. Its reported parameter count of only 0.09 M indicates that compact acoustic token design can retain strong diagnostic performance without excessive network depth.

Recent studies published in *Sensors* have demonstrated the value of smart sensor-based data acquisition for real-time bearing monitoring [11], acoustic-signal analysis and deep learning for bearing fault classification [12], cross-condition feature adaptation under varying operating conditions [13], and interpretable spectral evidence for bearing diagnosis. However, these approaches do not explicitly ensure that each acoustic frame represents a constant mechanical angle during continuously varying-speed operation. This gap motivates a synchronized acoustic–speed sensing framework in which mechanical-angle alignment precedes representation learning.

Despite this progress, fixed-duration acoustic frames lose their physical consistency when rotational speed varies. For a sampling frequency f_s_ and a frame length of L samples, the mechanical angle covered by a frame is proportional to the instantaneous speed. A frame of the same duration may therefore span different fractions of a shaft revolution at different times, while fault-related frequencies are stretched or compressed in the time domain. Consequently, fixed time windows may contain different stages of an impact response or even overlap adjacent impacts at different speeds. This kinematic inconsistency weakens the phoneme analogy: the tokens retain the same duration but no longer represent the same mechanical extent.

Order tracking addresses this problem by integrating the speed signal and resampling at equal angular intervals [14,15]. In the angle domain, components synchronized with shaft rotation are approximately stationary in order rather than fixed in frequency, providing a common representation for different speed trajectories. Recent physics-informed networks have incorporated speed information or learnable wavelet kernels into variable-speed fault diagnosis [16,17,18]. Most of these studies, however, focus on vibration signals, and the combination of angular normalization with amplitude–phase modeling of acoustic transients remains insufficiently explored. Directly transferring an existing acoustic frame embedding to the angle domain is also incomplete because the analytic representation itself may need to adapt when transmission paths, microphone responses, and resonance distributions change. The standard Hilbert transform provides a mathematically rigorous analytic signal with suppressed negative-frequency redundancy and an explicit quadrature component, but its response is fixed in advance. It cannot learn which order bands should be enhanced, attenuated, or phase-compensated for a specific acoustic diagnostic task. An unconstrained learnable filter is more flexible but may destroy the one-sided analytic structure and weaken channel interpretability. We therefore retain the Hilbert operator as an analytic prior and learn only bounded and smooth complex residual responses, with regularization limiting the departure from the physical initialization.

Interpretability is another unresolved issue. Temporal saliency maps can indicate where a model attends, but they do not directly identify the mechanical evidence supporting an inner-race, outer-race, rolling-element, or cage-fault decision. When a Kolmogorov–Arnold network (KAN) receives anonymous latent features, its learned functions cannot be directly associated with specific mechanical evidence. KANs replace fixed nodal activations with learnable univariate functions on network edges and can reveal sparse functional relationships [19,20]. Concept bottleneck models force the final decision to pass through human-readable concepts and permit test-time concept intervention [21,22]. Their combination is therefore most informative when the KAN inputs are physically defined concepts rather than unnamed latent variables.

Based on these considerations, this study proposes OSAP-KANFormer for bearing fault diagnosis using synchronized acoustic and rotational-speed measurements. Equal-angle resampling establishes a common mechanical scale before amplitude–phase representation learning. The final decision passes through predicted physical concepts and an additive KAN classifier. The complete architecture is presented in Section 2.4, while the interpretation and applicability of this decision pathway are discussed after the experimental results.

The main contributions of this study are summarized as follows:

(1) A synchronized acoustic and rotational-speed sensing representation is established for variable-speed bearing diagnosis. The measured speed signal is integrated to construct a shaft-angle map, and the acoustic waveform is resampled at equal angular intervals. Consequently, each sample and frame token covers a fixed mechanical extent rather than a fixed duration.
(2) A physics-constrained order-synchronous amplitude–phase learning architecture is developed. A Hilbert-initialized learnable analytic representation preserves the one-sided spectrum while introducing bounded and smooth amplitude and phase residuals. Reciprocal cross-attention subsequently models the complementary relationships between transient amplitude and circular-phase information.
(3) An explicit concept-mediated diagnostic pathway is constructed by combining physically named descriptors with a sparse additive KAN. The resulting class evidence can be inspected through concept-to-class functions and evaluated using matched contribution-ranking and intervention analyses. The inference pathway excludes a direct latent-feature bypass, while semantic fidelity is assessed separately from this architectural property.

## 2. Materials and Methods

### 2.1. Variable-Speed Kinematics and Equal-Angle Resampling

Let *x*(*t*) denote the measured acoustic observation. In Cases 1 and 3, *x*(*t*) is the airborne sound-pressure signal acquired by a microphone; in Case 2, it is the structure-borne acoustic channel acquired by a magnetically mounted sensor. Let *n*(*t*) denote the synchronously measured shaft speed in r/min. The cumulative shaft angle is obtained by integrating the speed, as given in Equation (1):

(1) $$\theta \left(t\right)=(2\pi /60)\int_{0}^{t}n(t)dt$$

A uniform angular grid is defined as *θ_m_* = *θ*_0_ + *m*Δ*θ*, where Δ*θ* = 2*π*/*N_r_* and *N_r_* is the number of resampled points per revolution. Monotonic interpolation of *θ*(*t*) provides the inverse mapping *t*(*θ_m_*), and the angle-domain waveform is expressed by the angular resampling relation in Equation (2):

(2) $$x\theta \left[m\right]=x\left(t\left(\theta_{m}\right)\right),m=0,1,\cdots ,T_{\theta }-1$$

For a sample containing k revolutions, *T_θ_* = *kN_r_*. The default settings are *N_r_* = 256 and *k* = 4, giving 1024 angular samples. A 16-point frame therefore always spans 16/256 = 0.0625 revolution, or 22.5°, irrespective of the instantaneous speed.

For a rolling bearing with *Z* rolling elements, rolling-element diameter *d*, pitch diameter *D_p_*, and contact angle *α*, the characteristic fault orders are defined in Equation (3):

(3) $$o_{BPFO}=\frac{Z}{2}(1-\frac{d}{D_{p}}cos\;\alpha )\;\;o_{BPFI}\;=\frac{Z}{2}\left(1+\frac{d}{D_{p}}cos\;\alpha \right)\;\;o_{FTF}\;\;\;=\frac{1}{2}\left(1-\frac{d}{D_{p}}cos\;\alpha \right)\;\;o_{BSF}\;\;\;=\frac{D_{p}}{2d}[1-{\left(\frac{d}{D_{p}}cos\;\alpha \right)}^{2}]$$

Unlike fault frequencies expressed in *Hz*, these orders remain approximately invariant with speed and therefore provide a natural coordinate system for both classification features and interpretable concepts.

Equal-angle resampling depends on the accuracy of the measured speed trajectory. Let $\tilde{n}\left(t\right)$ denote a perturbed speed measurement. Assuming the same initial angular reference, the resulting angular-mapping error is given in Equation (4):

(4) $$\delta \theta \left(t\right)=\frac{2\pi }{60}\int_{0}^{t}\left[\tilde{n}\left(u\right)-n\left(u\right)\right]\;du.$$

A persistent speed bias therefore produces an accumulating angular error. A synchronization offset introduces an additional mismatch between the acoustic observation and the speed trajectory, particularly during acceleration and deceleration. Missing encoder pulses affect the reconstruction of the speed trajectory and must be distinguished from additive perturbations applied to an already reconstructed speed signal.

### 2.2. Analytic Signal and Circular-Phase Representation

For the real-valued angle-domain signal $x_{\theta }\left[m\right]$, the Hilbert transform constructs the quadrature component ${\hat{x}}_{\theta }\left[m\right]$ and the corresponding analytic signal in Equation (5):

(5) $$z_{0}\left[m\right]=x_{\theta }\left[m\right]+j{\hat{x}}_{\theta }\left[m\right]=A_{0}[m]exp(j\phi_{0}[m])$$

The envelope magnitude $A_{0}\left[m\right]$ describes the local amplitude of the analytic signal, whereas $\phi_{0}\left[m\right]$ describes its oscillatory phase. A scalar phase representation contains an artificial jump between −π and π. The circular pair $\left(\cos\phi_{0},\sin\phi_{0}\right)$ avoids this discontinuity in the representation of absolute phase. Local phase evolution is additionally described by the wrapped phase increment in Equation (6):

(6) $$\Delta \phi_{0}\left[m\right]=\mathrm{Arg}\left(z_{0}\left[m\right]z_{0}^{*}\left[m-1\right]\right)$$

Here, Equation (6) is evaluated for $m=1,\ldots ,T_{\theta }-1$. ${\left(\cdot \right)}^{*}$ denotes complex conjugation and $Arg\left(\cdot \right)$ returns the principal angle in $\left(-\pi ,\pi \right]$. The product $z_{0}\left[m\right]z_{0}^{*}\left[m-1\right]$ therefore represents the phase difference between adjacent angular samples. For a sufficiently narrowband component with an unambiguous phase advance, $\Delta \phi_{0}\left[m\right]/\Delta \theta$ corresponds to instantaneous order. For broadband acoustic observations, it is treated as a phase-evolution descriptor rather than a direct estimate of a bearing characteristic fault order. The wrapped increment still has a principal-value boundary and is not described as globally discontinuity-free.

### 2.3. Concept Bottleneck and Additive KAN Evidence

A concept bottleneck decomposes the diagnostic mapping into a concept predictor *g* and a label predictor *f*, such that $\hat{y}=f(\hat{c})$ and $\hat{c}=g(x)$. The concepts are supervised by physical quantities that can be calculated or annotated independently of class labels [21]. Because the final prediction depends only on $\hat{c}$, an expert can replace a predicted concept with a counterfactual value and observe the resulting change in class probability. Compared with post hoc saliency alone, this procedure provides a more direct test of the model decision’s dependence on a concept.

A KAN replaces the fixed activations of a conventional multilayer perceptron with learnable univariate functions on the network edges [19]. For the compact concept vector, the class-wise additive KAN is written as the additive class-wise mapping in Equation (7):

(7) $$l_{k}=b_{k}+\sum_{m=1}^{M}f_{km}({\hat{c}}_{m})$$

where $f_{km}$ is the learnable evidence function connecting concept *m* to class *k*. After weak connections are removed through group sparsity, each logit can be expressed as the sum of a small number of concept contributions. Monotonicity constraints are applied only to mechanically justified relationships, such as the effect of BPFI-order energy on the inner-race-fault logit, rather than indiscriminately to all functions.

### 2.4. OSAP-KANFormer

#### 2.4.1. Overall Architecture

**Figure 1:**
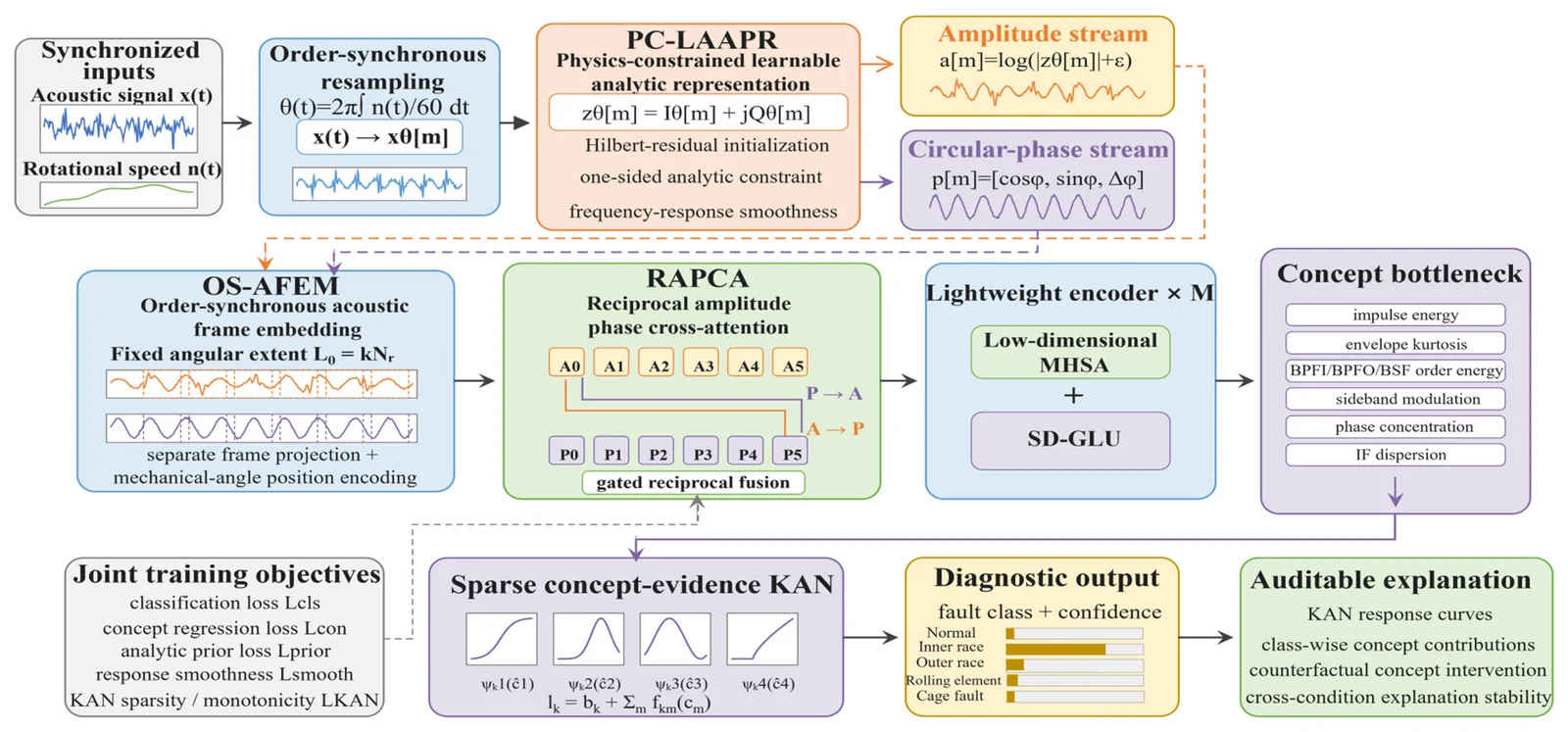
Overall architecture of OSAP-KANFormer.

Figure 1 presents the complete architecture of OSAP-KANFormer. The synchronized acoustic and rotational-speed measurements are first converted from the time domain to the shaft-angle domain through equal-angle resampling. PC-LAAPR then produces an amplitude stream and a circular-phase stream while preserving the analytic-signal structure. OS-AFEM converts the two streams into frame tokens with a fixed angular extent and a common embedding dimension. RAPCA performs reciprocal information exchange between the amplitude and phase tokens, after which a lightweight global–local encoder extracts long-range periodic dependencies and local transient patterns. The final class token is mapped to a set of physically named concepts, which are transformed into explicit concept-level class evidence by a sparse additive KAN. The auxiliary latent classifier is used during training and removed during inference. Consequently, the deployed class logits depend only on the predicted concept vector. This architectural restriction excludes a direct latent-feature bypass at inference.

#### 2.4.2. Physics-Constrained Learnable Analytic Amplitude Phase Representation (PC-LAAPR)

**Figure 2:**
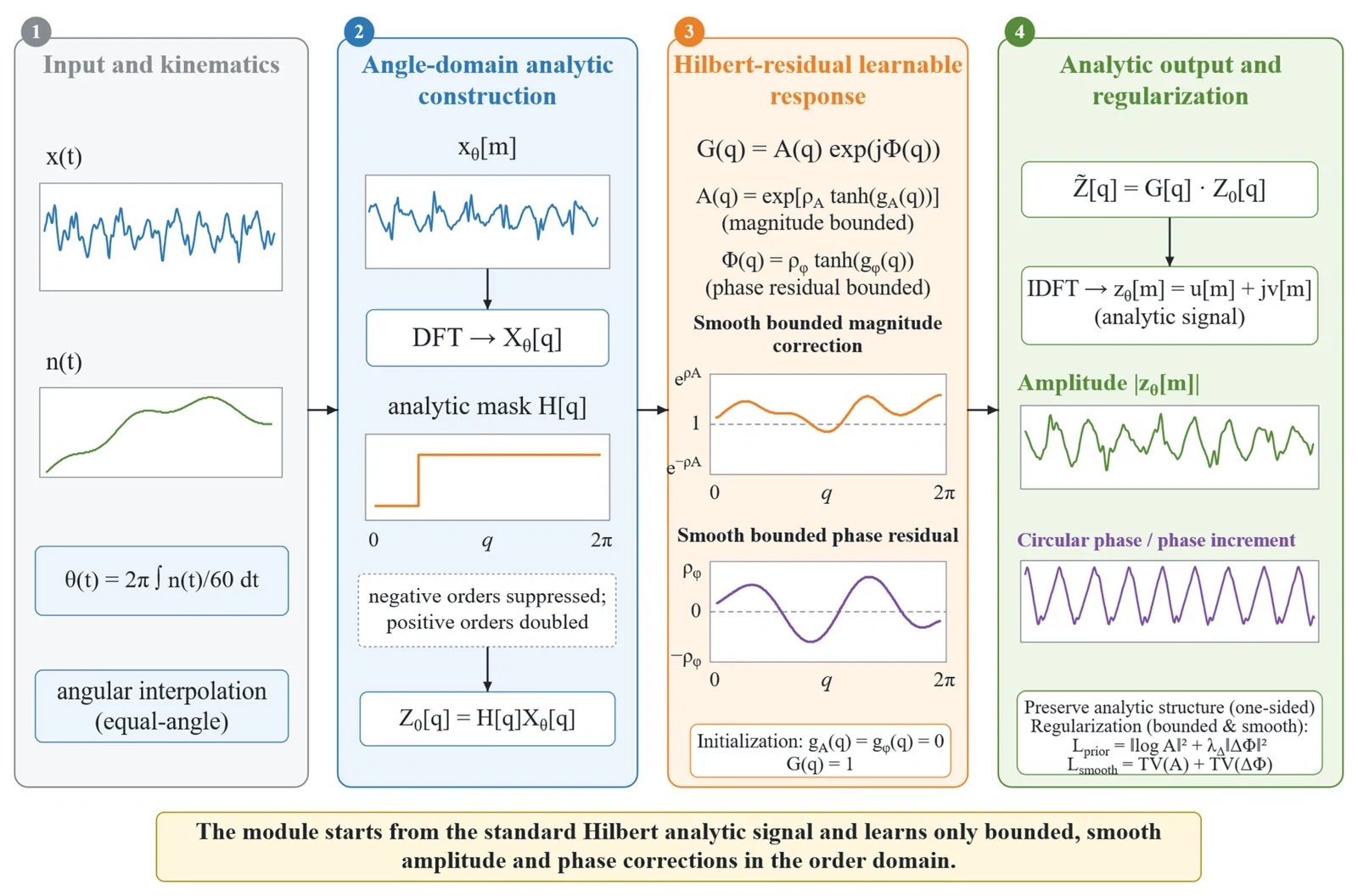
Physics-constrained learnable analytic amplitude phase representation (PC-LAAPR).

PC-LAAPR replaces the fixed AAPR used in the reproduced baseline, as illustrated in Figure 2. Let $X_{\theta }[q]$ denote the discrete Fourier transform of $x_{\theta }[m]$. The standard analytic mask $H_{a}[q]$ doubles the positive-order components, retains the direct-current and Nyquist terms, and suppresses negative-order components. A bounded learnable complex residual response is in Equation (8):

(8) $$G_{\Theta }\left[q\right]=A_{\Theta }[q]exp(j∆\phi_{\Theta }[q])$$

where $A_{\Theta }[q]$ is a positive amplitude response and $∆\phi_{\Theta }[q]$ is the phase residual. To ensure smoothness and limit the number of parameters, both responses are represented by q cubic *B*-spline basis functions $B_{q,r}$ in Equations (9) and (10):

(9) $$g_{A}\left[q\right]=\sum_{r}a_{r}B_{q,r}\;\;\;g_{\phi }\left[q\right]=\sum_{r}b_{r}B_{q,r}$$

(10) $$A_{\Theta }\left[q\right]\;\;\;=\exp\left[\rho_{A}\tanh\left(g_{A}\left[q\right]\right)\right]\;\;\Delta \phi_{\Theta }\left[q\right]=\rho_{\phi }tanhg_{\phi }\left[q\right]$$

The coefficients $a_{r}$ and $b_{r}$ are learnable, while $\rho_{A}$ and $\rho_{\phi }$ limit the maximum departure from the Hilbert prior. All coefficients are initialized to zero, giving $A_{\Theta }\left[q\right]$ = 1 and $\Delta \phi_{\Theta }\left[q\right]$ = 0 at the start of training. PC-LAAPR therefore begins exactly from the standard analytic output in Equation (11):

(11) $$Z_{\Theta }\left[q\right]=H_{a}\left[q\right]X_{\theta }\left[q\right]G_{\Theta }\left[q\right]\;\;\;z_{\Theta }\left[m\right]=IDFT\{Z_{\Theta }\left[q\right]\}$$

The one-sided spectrum is guaranteed directly by $H_{a}$ at the architectural level rather than indirectly through a penalty. The order domain output $z_{\Theta }\left[m\right]=I_{\Theta }\left[m\right]+jQ_{\Theta }[m]$ is then transformed into two streams, as shown in Equation (12):

(12) $$a\left[m\right]=log(\left|z_{\Theta }\left[m\right]\right|+\varepsilon )\;\;\;p\left[m\right]=\left[\frac{I_{\Theta }\left[m\right]}{\left|z_{\Theta }\left[m\right]\right|+\varepsilon },\frac{Q_{\Theta }[m]}{\left|z_{\Theta }\left[m\right]\right|+\varepsilon },\frac{\Delta \phi_{\Theta }\left[m\right]}{\pi }\right]$$

The logarithmic envelope compresses the dynamic range of high-energy impacts. The first two phase channels of $p\left[m\right]\;$ correspond to cosφ and sinφ, and the third describes local phase evolution. The learned order response can be plotted directly; a physically plausible model should produce smooth and bounded adjustments near diagnostically relevant resonance or order bands rather than irregular sample-to-sample oscillations.

#### 2.4.3. Order-Synchronous Acoustic Frame Embedding (OS-AFEM)

**Figure 3:**
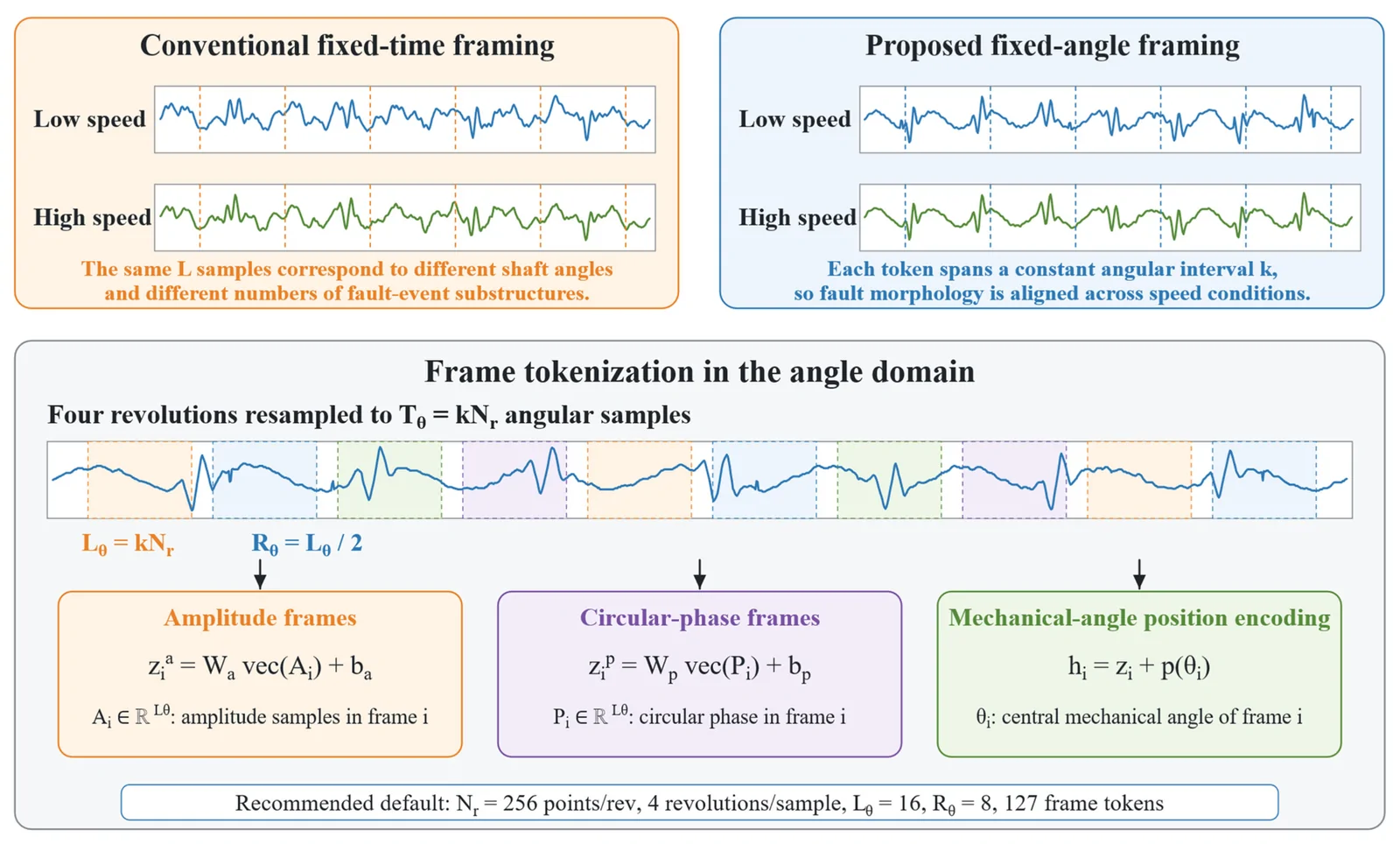
Order-synchronous acoustic frame embedding with a fixed angular extent (OS-AFEM).

As shown in Figure 3, OS-AFEM tokenizes the two angle-domain streams. For a sample containing $T_{\theta }$ points, a frame length $L_{\theta }$ and hop size $R_{\theta }$ produce the number of frames as shown in Equation (13):

(13) $$N=\left\lfloor \frac{T_{\theta }-L_{\theta }}{R_{\theta }}\right\rfloor +1$$

The *i*-th amplitude and phase frames are $A_{i}\in R^{1\times L_{\theta }}$ and $P_{i}\in R^{3\times L_{\theta }}$, respectively. Because the two streams have different statistical roles, independent linear projections are used in Equation (14):

(14) $$Z_{Ii}^{A}=W_{A}vec\left(A_{i}\right)+b_{A}\;\;\;Z_{Ii}^{P}=W_{P}vec\left(P_{i}\right)+b_{P}$$

For $T_{\theta }$ = 1024, $L_{\theta }$ = 16, and $R_{\theta }$ = 8, the number of frames is *N* = 127. Each token spans a fixed angle of 22.5°, and adjacent token centers are separated by 11.25°. A token-axis convolution with kernel size 5 in the SD-GLU covers 16 + 4 × 8 = 48 angular samples, corresponding to 67.5° of shaft rotation, independently of speed. This kinematic consistency distinguishes the method from fixed-time frame embedding.

A learnable class token is prepended to each stream. Mechanical-angle positional encoding is based on the frame-center angle normalized within the *k* revolution sample. A relative-angle bias is also introduced into attention, so token relationships depend on angular separation rather than elapsed time. The streams remain separate before RAPCA to avoid prematurely compressing their complementary amplitude and phase information through channel concatenation.

#### 2.4.4. Reciprocal Amplitude Phase Cross-Attention (RAPCA)

Standard unmasked self-attention can already access earlier and later tokens; therefore, reciprocal does not refer to forward and backward temporal visibility. In RAPCA, it denotes mutual information transfer between the amplitude and phase streams. Given *H_A_* and *H_P_*, the phase-to-amplitude and amplitude-to-phase contexts, respectively, are defined as shown Equation (15):

(15) $$C_{A\leftarrow P}=softmax(\frac{Q_{A}K_{P}^{T}}{\sqrt{d}})V_{P}\;\;\;C_{P\leftarrow A}=softmax(\frac{Q_{P}K_{A}^{T}}{\sqrt{d}})V_{A}$$

Each cross-stream context is modulated by a data-dependent gate in Equation (16):

(16) $$g_{A}=\sigma \left(W_{g_{A}}\left[H_{A};C_{A\leftarrow P}\right]\right)\;\;\;{\tilde{H}}_{A}=LN(H_{A}+g_{A}⊙C_{A\leftarrow P})\;\;g_{P}=\sigma \left(W_{g_{P}}\left[H_{P};C_{P\leftarrow A}\right]\right)\;\;{\tilde{H}}_{P}=LN(H_{P}+g_{P}⊙C_{P\leftarrow A})$$

**Figure 4:**
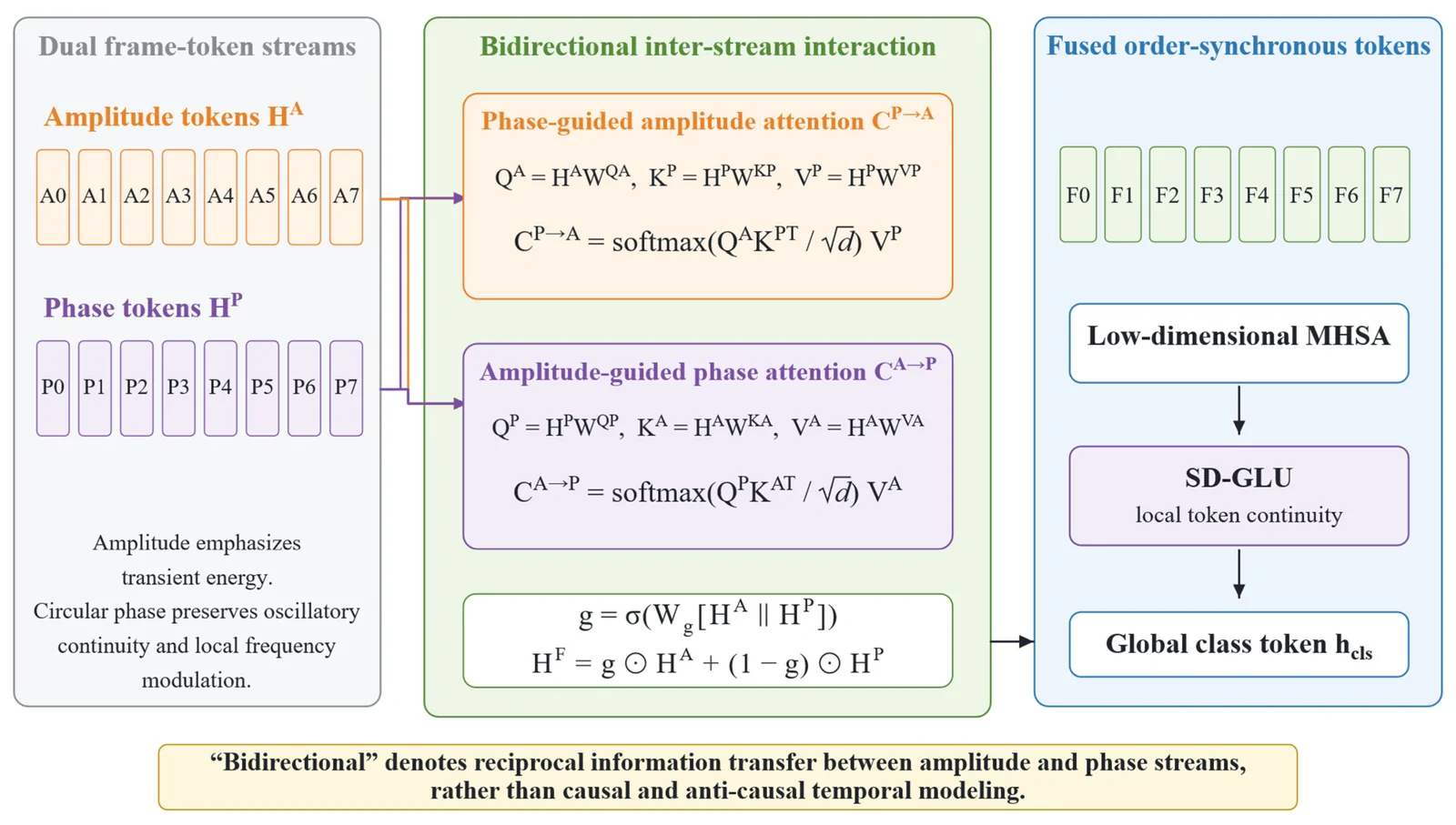
Reciprocal amplitude phase cross-attention and gated fusion (RAPCA).

A final fusion gate *η* combines the updated streams as $H_{F}=\eta ⊙{\tilde{H}}_{A}+(1-\eta )⊙{\tilde{H}}_{P}$. The mechanism uses phase coherence to screen amplitude transients with consistent periodicity, while amplitude energy emphasizes phase changes occurring during diagnostically relevant transients. As shown in Figure 4, To control complexity, RAPCA is used once before the encoder; deeper repeated cross-attention is evaluated only as a sensitivity variant.

#### 2.4.5. Lightweight Global Local Encoder

The fused tokens are processed by *M_enc_* encoder layers based on the efficient design of the reproduced ASFE-Transformer. Each layer contains low-dimensional MHSA and an SD-GLU feed-forward branch. The queries, keys, and values use compact per-head projection dimensions rather than equally dividing the full embedding dimension, reducing projection cost while retaining a global receptive field. The SD-GLU branch expands the channel dimension, applies SwiGLU gating, performs depthwise one-dimensional convolution along the token axis, and projects the features back to the embedding dimension. In the angle domain, this local convolution has a constant mechanical receptive field, so its inductive bias remains stable under speed variation, as given in Equation (17).

(17) $$Y^{\left(l\right)}=H^{\left(l-1\right)}+MHSA[LN(H^{\left(l-1\right)})]\;\;\;H^{\left(l\right)}=Y^{\left(l\right)}+SDGLU[LN(Y^{\left(l\right)})]$$

The final class token $h_{cls}$ aggregates information from the order-normalized acoustic sequence. For comparability with the original architecture, the default embedding dimension is 64, with four encoder layers, four attention heads, an SD-GLU expansion ratio of 2, and a depthwise-convolution kernel size of 5.

#### 2.4.6. Physical Concept Bottleneck and Sparse KAN Evidence

The concept layer predicts eleven named descriptors covering envelope energy, impulsiveness, spectral dispersion, characteristic-order energy, modulation, and circular-phase behavior. Each target is calculated from the corresponding sample without using its class label, and normalization parameters are fitted using the training set. The concept set is designed for the bearing configurations and fault categories evaluated in this study.

The eleven descriptors are not assumed to form a complete or minimally redundant representation of bearing damage. Some descriptors describe related properties: envelope kurtosis and crest factors both characterize impulsiveness, while characteristic-order energies and the sideband ratio capture related spectral structures. Group sparsity selects concept-to-class connections but does not establish that the input concept set is sufficient or free of redundancy. The effect of removing individual concepts or concept groups has not been quantified in this study.

A geometry-independent concept configuration is feasible in principle because envelope RMS, envelope kurtosis, crest factor, order-spectrum entropy, phase concentration, and phase-increment dispersion do not require the rolling-element count, rolling-element diameter, pitch diameter, or contact angle. These six descriptors could therefore be retained when reliable bearing geometry is unavailable. Characteristic-order energies and the current sideband-ratio definition would need to be omitted or redefined. Such a configuration would still require the rotational-speed reference for angular resampling and would constitute a separately trained concept predictor and classifier, rather than the existing model with selected inputs set to zero at inference. Its performance has not been evaluated here, and geometry independence should not be interpreted as independence from operating conditions, sensor characteristics, or bearing type.

The targets in Table 1 can be computed offline from each training sample, but the concept predictor is not intended to replace the underlying signal-processing formulas. Its role is to constrain the deep acoustic representation to an explicit physical-concept space so that the final classifier cannot rely directly on anonymous latent features. The concept indicators are calculated as supervision targets during training; at inference, the model predicts them directly from the class token without repeating the complete hand-crafted feature-extraction procedure.

To prevent indirect class-label leakage into the concept targets, every concept is calculated from an individual acoustic sample without using its class, predicted label, or test-set statistics. The BPFI, BPFO, BSF, and FTF orders are determined only by bearing geometry, and concept-scaling parameters are fitted only on the training set.

In Table 1, the band-energy aggregation function is defined as given in Equation (18):

(18) $$B\left(o_{c}\right)=\sum_{o\in [o_{c}-\delta ,o_{c}+\delta ]}E\left(o\right).$$

where E(o) is the order-spectrum energy, $o_{c}$ is the center order, and δ is the half-bandwidth.

For the sideband ratio $c_{9}$, the aggregated sideband energy $E_{sb}$ and center-order energy $E_{ctr}$ are defined as given in Equation (19):

(19) $$E_{sb}=\sum_{r\in O}\sum_{h=1}^{H}\left[E\left(hr-\Delta_{s}\right)+E\left(hr+\Delta_{s}\right)\right],\;\;\;E_{ctr}=\sum_{r\in O}\sum_{h=1}^{H}E\left(hr\right),$$

where $O=\left\{o_{BPFI},o_{BPFO},o_{BSF},o_{FTF}\right\}$ is the set of candidate characteristic orders, H  is the number of harmonics included, $\Delta_{s}$ is the sideband spacing in the order domain, and ε is a small positive constant used for numerical stability. The two aggregate quantities are evaluated over all candidate characteristic orders and their predefined harmonics. The center orders are determined from bearing geometry and are not selected using either the true class or the predicted class.

The concept predictor outputs the normalized continuous vector ${\hat{c}}_{m}=g_{c}(h_{cls})$. Because several impulsiveness indicators have heavy-tailed distributions, a Smooth-L1 concept loss is used. During inference, each class logit depends only on ${\hat{c}}_{m}$ through the additive KAN, as given in Equation (20):

(20) $$f_{km}\left(c\right)=\alpha_{km}c+\sum_{r=1}^{R}a_{kmr}B_{r}\left(c\right)\;\;\;l_{k}=b_{k}+\sum_{m=1}^{M}f_{km}({\hat{c}}_{m})$$

Group sparsity is imposed on each complete concept-to-class function so that weak connections can shrink to zero. A soft monotonicity constraint is applied only to the predefined mechanically matched set *S_mono_*, including inner race-BPFI energy, outer race-BPFO energy, rolling element-BSF energy, and cage-FTF energy, as given in Equation (21):

(21) $$L_{mono}=\sum_{(k,m)\in S_{mono}}mean[ReLU(-\frac{\partial f_{km}({\hat{c}}_{m})}{\partial {\hat{c}}_{m}})]$$

For a given sample, the contribution of concept *m* to class *k* is $E_{km}=f_{km}({\hat{c}}_{m})$. These signed values support concept-contribution plots, while the complete functions show how evidence changes with concept value. Counterfactual intervention replaces one concept with a value from a healthy or fault range and recomputes the class probabilities. Because there is no latent-feature bypass during inference, the prediction change can be attributed to the intervened concept path within the trained model.

#### 2.4.7. Joint Optimization and Training Strategy

The total loss is as given in Equation (22):

(22) $$L=L_{cls}+\lambda_{c}L_{con}+\lambda_{p}L_{prior}+\lambda_{s}L_{smooth}\;\;\;+\lambda_{sp}L_{sparse}+\lambda_{m}L_{mono}+\lambda_{aux}L_{aux}$$

where $L_{cls}$ is the cross-entropy loss on the KAN output; $L_{con}$ is the concept-regression loss; $L_{prior}$ penalizes departure from the Hilbert initialization; $L_{smooth}$ constrains the first-order variation in the learned response along the order axis; $L_{sparse}$ is the group-sparsity loss on the concept-to-class functions; $L_{mono}$ is defined in Equation (21); and $L_{aux}$ is an auxiliary latent-classification loss used only during training. The default weights are $\lambda_{c}$ = 0.5, $\lambda_{p}$ = 1 × 10^−3^, $\lambda_{s}$ = 5 × 10^−4^, $\lambda_{sp}$ = 1 × 10^−4^, $\lambda_{m}$ = 5 × 10^−3^, and $\lambda_{aux}$ = 0.2. Their sensitivity is examined in Section 3.6.

The auxiliary classification loss influences the shared encoder and can therefore change both concept prediction and downstream classification. Its effect must be distinguished from the structural restriction imposed by the concept bottleneck. Removing the auxiliary head at inference does not reverse its influence on representation learning. Moreover, removing the auxiliary loss alone does not eliminate all class-dependent gradients, because the main classification loss also propagates through the concept predictor during joint optimization.

Training is conducted in three stages with no more than 80 epochs. Stage 1 is a 15-epoch representation and concept warm-up. PC-LAAPR, OS-AFEM, RAPCA, the global local encoder, the concept predictor, and the auxiliary latent classifier are jointly optimized. The cubic-spline residual coefficients of Concept-KAN are fixed at zero, while the class biases and linear basis coefficients remain trainable, keeping the initial concept-to-class mapping approximately linear. This stage uses the classification, concept-regression, auxiliary-classification, analytic-prior, and smoothness losses without pruning concept-to-class edges.

Stage 2 performs full-model joint optimization for at most 50 epochs. The Concept-KAN spline coefficients are unfrozen, and all modules are optimized using Equation (22). The initial learning rate is 1 × 10^−3^, followed by five epochs of linear warm-up and cosine decay to a minimum of 1 × 10^−5^. Model selection is based only on validation Macro-F1. If validation Macro-F1 does not improve for 12 consecutive epochs, Stage 2 is terminated early, and the best validation checkpoint is restored.

Stage 3 performs sparse pruning and fine-tuning for 15 epochs. For the evidence function $f_{km}$ from concept *m* to class *k*, its normalized functional amplitude is first evaluated over the 1st–99th percentile range of the training-set concept values, as given in Equation (23):

(23) $$r_{km}=\frac{{\left[\frac{1}{G}\sum_{g=1}^{G}{\left(f_{km}(c_{m}^{\left(g\right)})-{\bar{f}}_{km}\right)}^{2}\right]}^{1/2}}{\underset{j}{max}{\left[\frac{1}{G}\sum_{g=1}^{G}{\left(f_{kj}(c_{j}^{\left(g\right)})-{\bar{f}}_{kj}\right)}^{2}\right]}^{1/2}+\varepsilon }$$

Here, *G* = 100 is the number of uniformly spaced concept-grid points, and *ε* = 10^−8^ ensures numerical stability. When $r_{km}$ < 0.05, the linear coefficient $\alpha_{km}$ and all spline coefficients $\alpha_{kmr}$ of that connection are set to zero and frozen. The pruned model is fine-tuned for 15 epochs with a learning rate of 1 × 10^−4^, and removed connections are not reactivated. The final checkpoint is selected by validation Macro-F1 during fine-tuning; the test set is not used for model selection, pruning-threshold determination, or early stopping.

The quantity $r_{km}$ measures the normalized variation in a concept-to-class function over the training-supported concept range. A threshold of 0.05 therefore identifies connections whose functional variation is small relative to the largest variation within the same class. It is a pruning parameter, not a significance level or a threshold on class probability. Its effect is evaluated separately from that of the group-sparsity regularization weight.

#### 2.4.8. Diagnostic and Explanation Workflow

For each source record, the acoustic observation and speed signal are first synchronized and calibrated. The record is assigned to the training, validation, or test set before segmentation. Each segment is converted to the angle domain and processed by OSAP-KANFormer to obtain a fault class. The model also outputs predicted concepts, class-wise KAN contributions, probabilities calibrated by validation-set temperature scaling, and optional counterfactual results. Evaluation therefore extends beyond classification accuracy by linking each prediction to explicit mechanical evidence.

### 2.5. Experimental Validation

#### 2.5.1. Datasets and Sensing Configurations

Three complementary cases are considered. Case 1 reproduces a fixed-speed acoustic benchmark for architectural comparison. Case 2 uses synchronously acquired structure-borne acoustic and rotational-speed measurements from a cold-rolling production line to evaluate order-synchronous modeling. Case 3 uses an external multi-condition airborne-acoustic dataset to assess transfer across speed, load, and microphone conditions [23]. Airborne sound-pressure signals acquired by microphones and structure-borne acoustic signals acquired by magnetically mounted sensor nodes are collectively referred to as acoustic sensing signals only when describing the common diagnostic pipeline. Their physical quantities, transmission paths, calibration procedures, and measurement units are treated separately and are not assumed to be equivalent.

In Table 2, Case 1 follows the basic configuration of the reproduced study: the input records contain ten bearing conditions, with a nominal speed of 900 r/min, a radial load of 5 kN, and an acoustic sampling rate of 20 kHz. The principal difference from the original study is the data split. Instead of generating 50 overlapping windows, randomly shuffling them, and allocating windows across subsets, continuous source records are first divided into non-overlapping time blocks or independent acquisition batches before any model window is formed.

Case 2 was collected at a key rolling-bearing housing in the main-drive system of a cold-rolling production line. The equipment experiences start-up, acceleration, steady rolling, speed adjustment, deceleration, and shutdown, thereby covering representative continuous varying-speed operation. Five conditions are considered: healthy, inner-race fault, outer-race fault, rolling-element fault, and cage fault. Fault labels were determined from inspection records, maintenance and replacement records, and shutdown disassembly; the acoustic signal was not used to generate labels.

**Figure 5:**
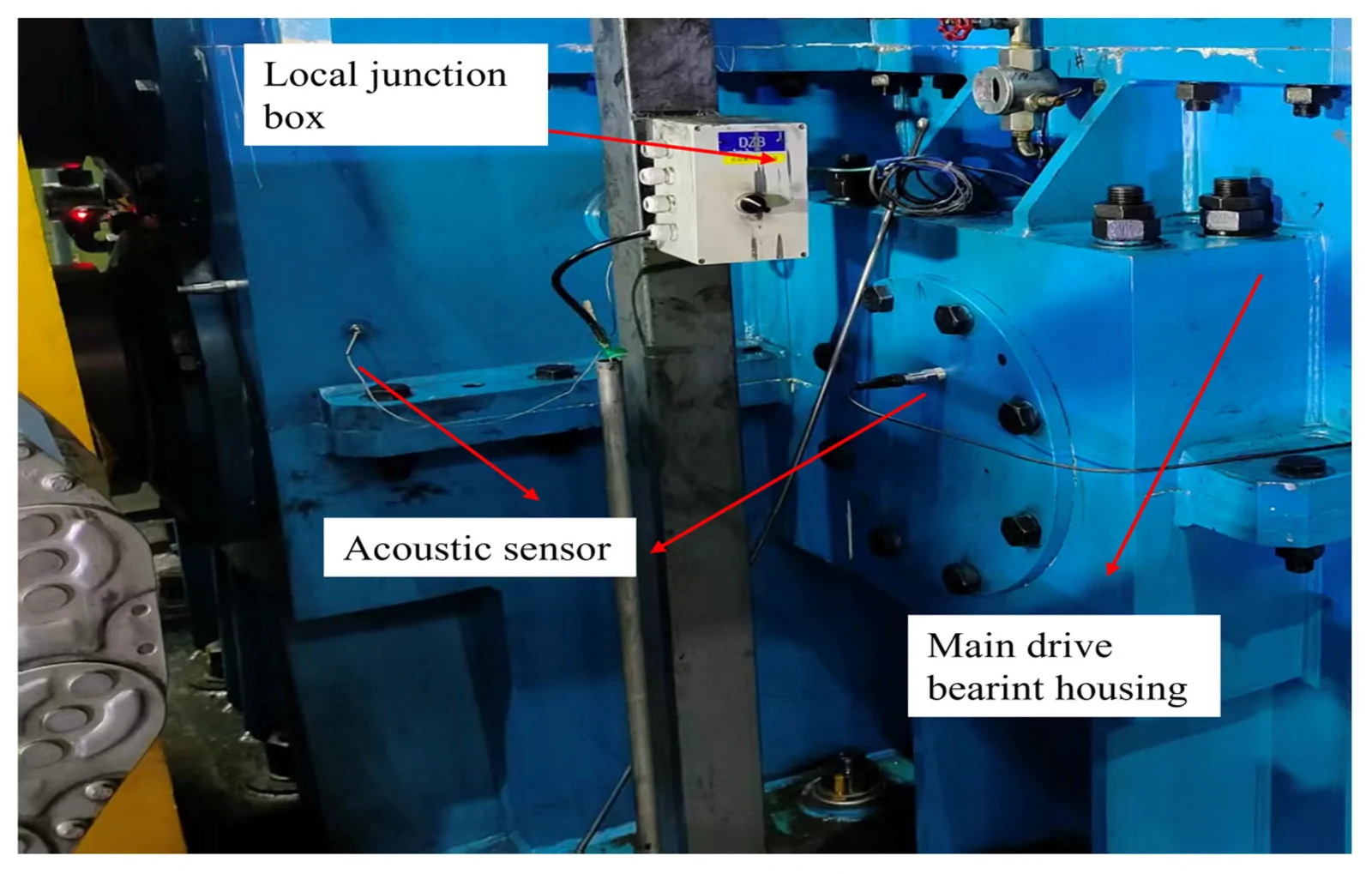
Field data-acquisition area for Case 2.

Figure 5 shows the measurement area in the cold-rolling main-drive system, including the bearing housing, the mounted acoustic sensor, and the local junction box. The photograph provides the installation context for the structure-borne acoustic measurements used in Case 2.

**Figure 6:**
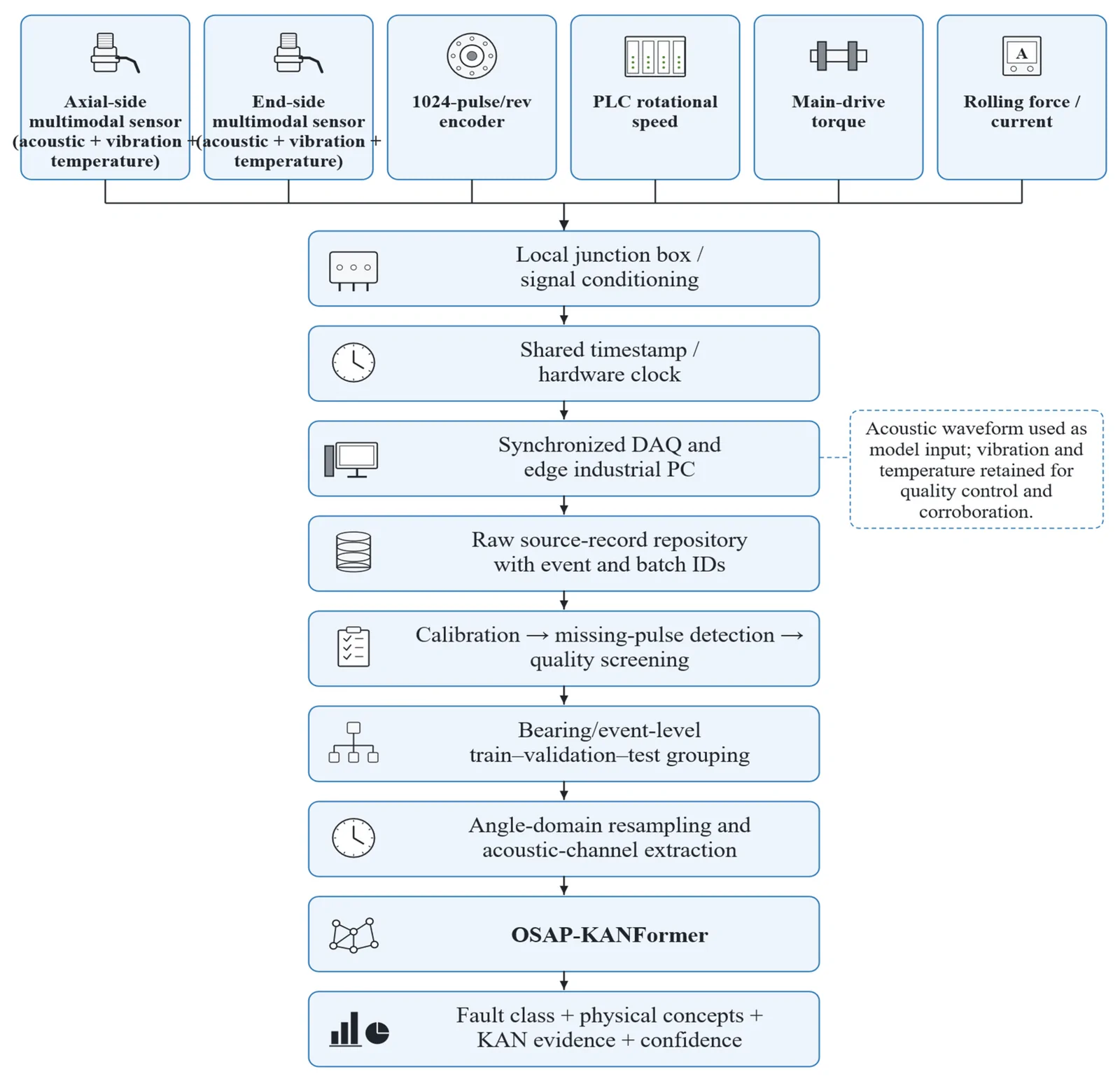
Synchronized field data-acquisition chain for the cold-rolling main drive system.

As shown in Table 3, The cold-rolling field dataset contains 32 independent bearing entities or maintenance-confirmed events. After quality screening, 175 synchronized source records from 91 production batches were retained. Healthy, inner-race-fault, outer-race-fault, rolling-element-fault, and cage-fault conditions contained 72, 29, 28, 23, and 23 source records, respectively. Source records are allocated before segmentation. Under P1, the held-out speed is excluded from training and validation, while different recordings from the same bearing entity may occur at training and test speeds. P1 therefore evaluates unseen-speed generalization for previously observed bearing entities. Generating windows only after source-record allocation prevents overlapping windows from the same underlying recording from entering different subsets; it does not establish bearing-entity independence. Overlapping segments were generated only after source-record allocation, preventing the same physical event from leaking across subsets. Records affected by missing speed pulses, sensor saturation, signal interruption, or incomplete batch information were excluded during quality screening. Because field fault samples were imbalanced, the classification loss used inverse-frequency class weights calculated only from the number of training source records. The validation and test sets retained their original class distributions without over- or under-sampling. The IN-VCM-N225 sensor(made in China, Tianjin) provides acoustic, temperature, and vibration channels, uses magnetic mounting, and records the acoustic waveform at 51.2 kHz. The complete acquisition chain is shown in Figure 6.

The field measurement subsystem used two IN-VCM-N225 magnetically mounted multimodal sensor nodes installed at the axial-side and end-side positions of the main-drive bearing housing. The structure-borne acoustic channel was sampled at 51.2 kHz. Shaft speed was obtained from a 1024-pulse-per-revolution encoder. Acoustic, rotational-speed, and PLC measurements were aligned using the shared hardware timestamp and stored as synchronized source records on an edge industrial computer. Only the acoustic waveform and the speed reference required for angular mapping were supplied to the diagnostic pipeline.

The structure-borne acoustic channel represents sensor-output voltage in volts (V) over a calibrated usable frequency range of 50 Hz–20 kHz. The recorded signal is converted using a unity-gain voltage-conditioning stage (1.0 V/V) and a nominal ADC conversion coefficient of 2.9802 × 10^−7^ V/count, and the acquisition resolution is 24 bits over a ±2.5 V bipolar input range at a sampling rate of 51.2 kHz. Acoustic–speed synchronization accuracy is a residual timing offset of no more than 10 μs, determined from a common timing event with channel-dependent delay compensation.

Only the acoustic waveform was used as the diagnostic signal input to OSAP-KANFormer. The rotational-speed measurement was used exclusively to construct the shaft-angle map and perform equal-angle resampling. Vibration and temperature channels were retained for signal-quality assessment, operating-state verification, and maintenance-label corroboration, while the PLC variables were used as operating-load proxies. Thus, the classification model did not use vibration, temperature, current, or load variables as additional diagnostic inputs. Data allocation followed the protocol-specific grouping rules in Section 2.5.2 and was completed before signal segmentation.

Case 3 uses a public multi-condition acoustic dataset containing three nominal speeds (800, 1000, and 1200 r/min), three load levels (0%, 15%, and 30% of rated torque), and three synchronized microphones. Each operating condition was recorded for 50 min at 32,768 Hz. These long continuous records support file-level splitting and cross-microphone evaluation. The healthy-bearing and cage-fracture subsets were selected to construct binary cross-load, cross-microphone, and compound-shift tasks. This selection retains a consistent fault definition while focusing on transfer across sensor and operating-condition domains.

#### 2.5.2. Leakage-Free Data Splits and Evaluation Protocols

Data partitioning is completed before segmentation. The protected grouping unit is specified separately for each evaluation protocol. No raw acoustic sample or simultaneously acquired multisensor interval is shared between training, validation, and testing. Adjacent time blocks are separated by a guard interval sufficient to prevent overlapping between model observation windows. Normalization, concept scaling, and class weighting are fitted using the training partition, while model selection and probability calibration use the validation partition. Test labels are used only for performance evaluation.

In P1, the held-out speed is excluded from both training and validation, but different source records from the same bearing entity may occur at training and test speeds. P1 therefore evaluates generalization to unseen speeds for previously observed bearing entities. It does not establish simultaneous generalization to unseen speeds and unseen bearings. Table 4 shows the Cross condition evaluation protocols.

For constant-speed records, the known nominal speed is sufficient for equal-angle resampling. For variable-speed records, the synchronous speed trajectory is low-pass filtered only to suppress encoder quantization noise, constrained to remain positive, and then integrated according to Equation (1). The inverse mapping t(θ) is obtained by cubic monotonic interpolation. Samples affected by missing speed pulses or falling outside the valid angular-mapping range are excluded and reported. Under P3, the three synchronized microphone channels from the same operating time block are assigned together to a single subset, so a test time block cannot enter training through another microphone channel.

#### 2.5.3. Comparison Methods and Implementation Details

The proposed model is compared with conventional methods, Transformer-based methods, physics-informed methods, and KAN-related baselines. All compared methods use the same downstream source-record partitions. External pre-training is distinguished from downstream task training, and identical total pre-training data or computational budgets are not assumed. Input representations are unified when feasible, and parameter-matched variants are included to distinguish architectural effects from model-capacity effects. The three datasets evaluate the applicability of the framework to different sensing configurations and diagnostic tasks. They do not constitute zero-shot transfer of a single trained model across datasets. Model weights, classification heads, and probability-calibration parameters are fitted separately for each dataset. Geometry-dependent concepts are configured using the available bearing information. Accordingly, the reported cross-condition results describe transfer between operating conditions within a dataset, rather than deployment across the three datasets without re-training. This study does not evaluate whether a single hyperparameter configuration can be transferred across all three datasets without dataset-specific selection.

For the fixed-speed data in Case 1, all methods use acoustic segments of the same duration. For the variable-speed data in Case 2, samples for all methods are generated from the same synchronized acoustic–speed source records. The classification head is reinitialized according to the number of classes in each dataset. Case 2 uses five outputs-healthy, inner-race fault, outer-race fault, rolling-element fault, and cage fault, whereas Case 3 uses only healthy and cage-fracture outputs. The physical concepts in Concept-KAN are configured according to the bearing geometry available for each dataset. Parameter counts and FLOPs are recalculated for the actual output layer and concept configuration. Performance values are compared only within the same dataset and protocol; absolute Macro-F1 values are not compared across datasets. Table 5 presents the baseline and ablation oriented comparison methods.

The architecture and training procedure of the proposed OSAP-KANFormer are described separately in Section 2.4 and Table 6. BEATs and SSAMBA were initialized from BEATs_iter3+ (AS2M) and SSAMBA-base (400 masked patches), respectively, using their official PyTorch 2.5.1 implementations. Acoustic intervals corresponding to the four-revolution samples were resampled to 16 kHz and converted into 128-bin filterbank features using 25 ms windows and 10 ms frame shifts, with model-specific normalization. BEATs used batchwise padding, padding masks, and masked temporal pooling; SSAMBA used right-padding to 128 frames and average token pooling. Both encoders were fully fine-tuned with newly initialized five-class heads comprising layer normalization, dropout (0.1), and a linear classifier. Optimization used AdamW, encoder and classifier learning rates of 1 × 10^−5^ and 1 × 10^−4^, respectively, weight decay of 0.01, and an effective batch size of 64. Training lasted at most 80 epochs, with 5 warm-up epochs, cosine learning-rate decay, and early stopping patience of 12 epochs. Checkpoints were selected by validation Macro-F1 before testing. Table 6 shows the Default implementation and hyperparameter settings.

To prevent concept targets from introducing test-set statistics, each continuous concept is winsorized and robustly scaled using the 1st, 50th, and 99th percentiles estimated from the training set. When microphone-sensitivity metadata are available, acquisition voltage is converted to sound pressure. Test samples are not normalized by their own global amplitude because this operation would remove information needed by the energy and crest-factor concepts. Instead, sensor calibration and robust normalization fitted only on the training set are used. All models are probability-calibrated by temperature scaling on the validation set. The temperature is obtained by minimizing validation negative log-likelihood. Expected calibration error (ECE) is calculated with 15 equal-width confidence bins, and the test set is not used to fit the calibration parameter [26].

#### 2.5.4. Evaluation Metrics and Statistical Analysis

Because cross-condition subsets may be class-imbalanced, Macro-F1 is the primary classification metric. Accuracy and ECE are also reported for fixed-condition experiments, and class-wise F1 scores are reported for the core field experiment. Efficiency is evaluated by the number of trainable parameters, FLOPs, preprocessing time, network inference time, total computational latency, and peak memory. Concept-prediction quality is assessed using normalized mean absolute error (MAE), the coefficient of determination (R^2^), and the Pearson correlation coefficient.

Explanation analysis covers concept-prediction quality, active concept-to-class connections, learned evidence functions, cross-seed contribution-ranking stability, and a representative concept-intervention example. Cross-seed contribution-ranking stability is quantified by Spearman correlation using the common concept-intervention definition described below. Concept-KAN and the concept MLP use the same contribution definition: for a given sample, target class, and concept, the contribution is the change in the target-class logit after replacing that concept with a training-derived reference, taken as the healthy-training median. For the additive KAN, this is simply the difference between the learned univariate function at the predicted concept value and at the reference value. Within each source record, absolute contributions are averaged over test windows and ranked across concepts, and rankings are compared between seeds using the same held-out records and target class. The true class is used only to fix the target of this post-training evaluation. Spearman correlation is computed per eligible record and seed pair and then averaged; tied ranks receive average ranks, and constant ranking vectors yield undefined correlations and are reported separately. The ten pairs from five seeds are not treated as ten independent experiments. This value summarizes cross-seed reproducibility under the specified protocol and does not measure physical causality or expert usefulness. The main classification, robustness, and sensitivity metrics are reported as the mean ± standard deviation over five independent runs. Confusion matrices, ablation changes, and representative explanation cases are reported from aggregated test results or representative source records.

Spearman correlation is calculated for each eligible source-record–fold unit and seed pair. In addition to the averaged correlation, the distribution of the individual valid coefficients is summarized using the median, interquartile range, and 5th–95th percentiles. These quantiles are calculated before averaging over records or seed pairs. The number of eligible record–fold units, the number of valid coefficients, and the number of undefined coefficients are reported explicitly. Tied ranks receive average ranks, and constant ranking vectors yield undefined correlations rather than zero correlation. Coefficients sharing source records or trained models are treated as dependent descriptive observations.

## 3. Results and Discussion

### 3.1. Effect of Strict Splitting and Sensor Conditions on Acoustic Characteristics

Under the original random window-level split, the reproduced ASFE-Transformer achieved an accuracy of 99.62 ± 0.11%, which differs from the reported 99.75% by 0.13 percentage points. Under the strict source-record-level protocol, its accuracy and Macro-F1 decreased to 96.8 ± 0.6% and 96.5 ± 0.7%, respectively. The 2.82-percentage-point accuracy difference between window-level and record-level evaluation shows that splitting overlapping windows can overestimate generalization performance.

As shown in Table 7, Under the strict protocol, OSAP-KANFormer achieved the highest Macro-F1 of 97.3 ± 0.5%, exceeding the 96.5 ± 0.7% of ASFE-Transformer by 0.8 percentage points. Thus, introducing order-synchronous amplitude phase representation and a concept-evidence path did not reduce fixed-speed diagnostic performance. The following cross-condition experiments evaluate generalization to unseen speeds, loads, and sensor conditions. Two magnetically mounted multimodal sensors were installed at the axial-side and end-side positions in the field, enabling the influence of structure-borne acoustic transmission paths to be compared between channels. The results are listed in Table 8.

Sensor position and operating stage affected the measured acoustic characteristics. In this installation, the axial-side channel showed higher characteristic-band SNR than the end-side channel, while transient operating stages showed lower spectral consistency than steady rolling. Order synchronization reduced speed-related feature displacement but did not eliminate differences in structural transmission paths.

These results show that order synchronization does not eliminate the influence of the sensing path. The axial-side and end-side nodes exhibited different characteristic-band SNRs and fault-to-healthy energy contrasts because the measured structure-borne acoustic response depends on mounting orientation and structural transmission. Order synchronization improves the consistency of fault-feature locations across speeds, but it cannot make different sensor transfer functions identical. Therefore, the axial-side position should not be assumed to be superior for every cold-rolling installation.

### 3.2. Generalization to Unseen Speeds and Speed Trajectories

Table 9 summarizes the unseen-speed and trajectory results. When the test speed was absent from training, the fixed-time ASFE tokens degraded because they covered different angular extents at different speeds. Adding only equal-angle resampling increased leave-one-speed-out Macro-F1 by 4.6 percentage points, the largest single-step gain in the cumulative-module experiment. PC-LAAPR and RAPCA provided further improvement through adaptive order-domain amplitude phase representation and complementary cross-stream information. Protocol P1 contains four held-out-speed folds, with 600, 900, 1200, and 1500 r/min used in turn as the unseen test speed. Each fold was evaluated with five random seeds. The four folds were first averaged within each seed, after which the mean and standard deviation were calculated across the five seeds. Source-record grouping was repeated in every fold. Records from the same bearing entity at different speeds were allowed to enter the training and test sets, so P1 evaluates unseen-speed generalization for previously observed entities. The test set contains only the predefined held-out speed, while training and validation use the remaining speeds.

As shown in Table 9 and Table 10, for the leave-one-speed-out evaluation, OSAP-KANFormer achieved a Macro-F1 of 92.4 ± 1.2%, 7.3 percentage points above the 85.1 ± 1.8% of ASFE-Transformer. For constant-speed-to-variable-speed transfer, the proposed method achieved 90.5 ± 1.5%, exceeding ASFE-Transformer by 9.2 percentage points. Performance and stability declined as the evaluation progressed from unseen speed to unseen variable-speed trajectories and the most difficult compound sweep trajectory. BEATs achieved the highest mean Macro-F1 under leave-one-speed-out evaluation, with 92.6% compared with 92.4% for OSAP-KANFormer. The proposed method obtained higher mean performance in constant-speed-to-variable-speed transfer and worst-trajectory evaluation and tied SSAMBA in mean leave-one-variable-speed-trajectory-out performance. These differences are descriptive and are not interpreted as statistically significant without a corresponding paired analysis.

**Figure 7:**
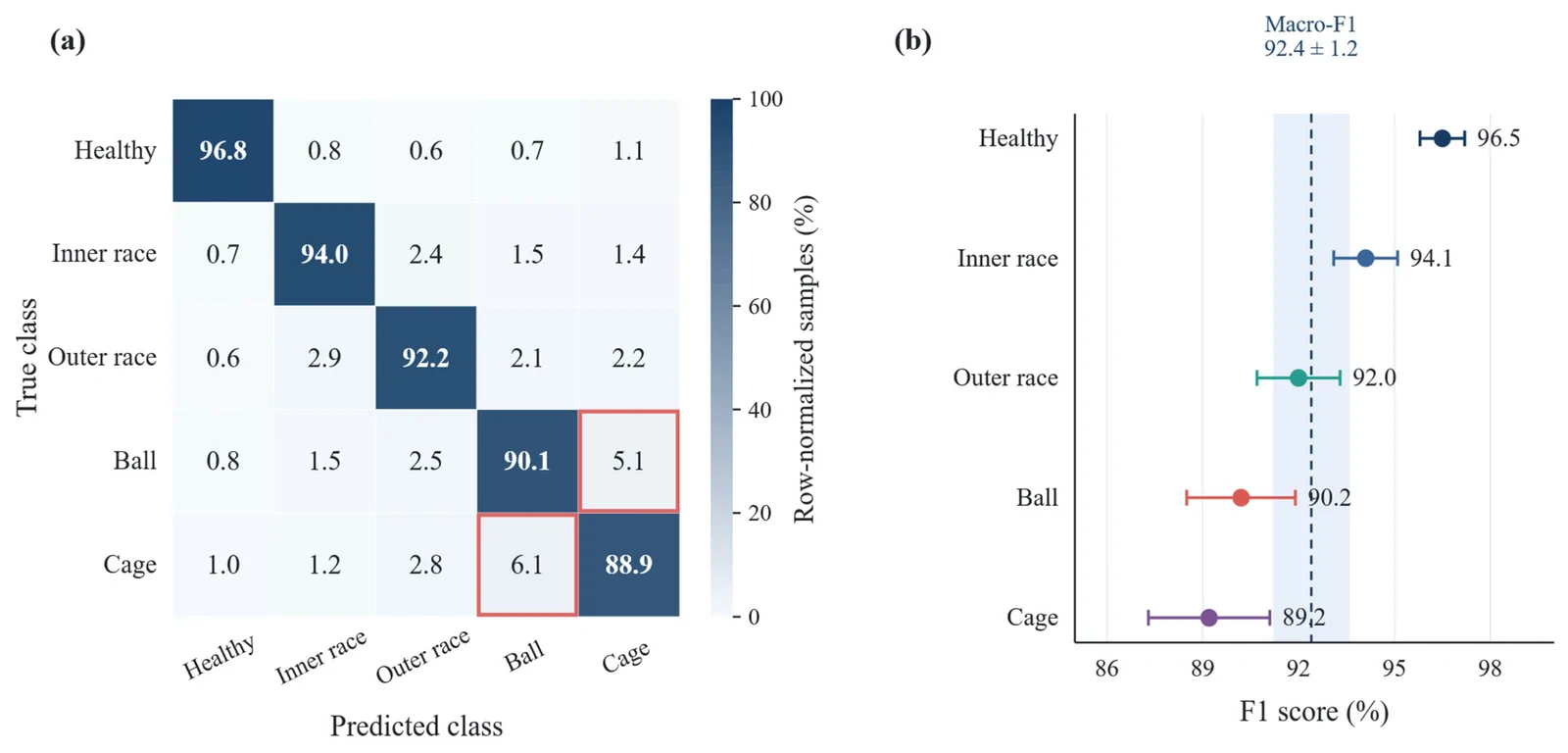
Confusion matrix and class-wise F1 scores. (**a**) Confusion matrix (**b**) Class-wise performance.

Figure 7 shows stable recognition of healthy, inner-race-fault, and outer-race-fault conditions, with recalls above 92%. The largest confusion occurred between rolling-element and cage faults: 5.1% of rolling-element-fault samples were classified as cage faults, and 6.1% of cage-fault samples were classified as rolling-element faults. Both faults can produce broadband impacts modulated by cage motion and may exhibit similar sideband structures under low main-drive torque and rapid speed sweeps.

A small amount of bidirectional confusion remained between inner- and outer-race faults, but it was substantially lower than the rolling-element-cage confusion, indicating that BPFI- and BPFO-order concepts provide comparatively stable location evidence. Cage faults had the lowest class-wise F1, so the subsequent failure-case and concept analyses focused on weak FTF energy and its competition with BSF-related modulation.

To quantify the effect of speed variation on fault-feature alignment, four metrics were calculated:

1. Peak-alignment error: the absolute difference between the observed peak order and the theoretical fault order.
2. Characteristic-band concentration ratio: the proportion of total order-domain energy located around the fault order and its first three harmonics.
3. Cross-speed cosine similarity: the mean cosine similarity between envelope-order spectra from different speed ranges.
4. Characteristic-band SNR: the ratio of energy in the fault-order band to that in adjacent background-order bands.

**Figure 8:**
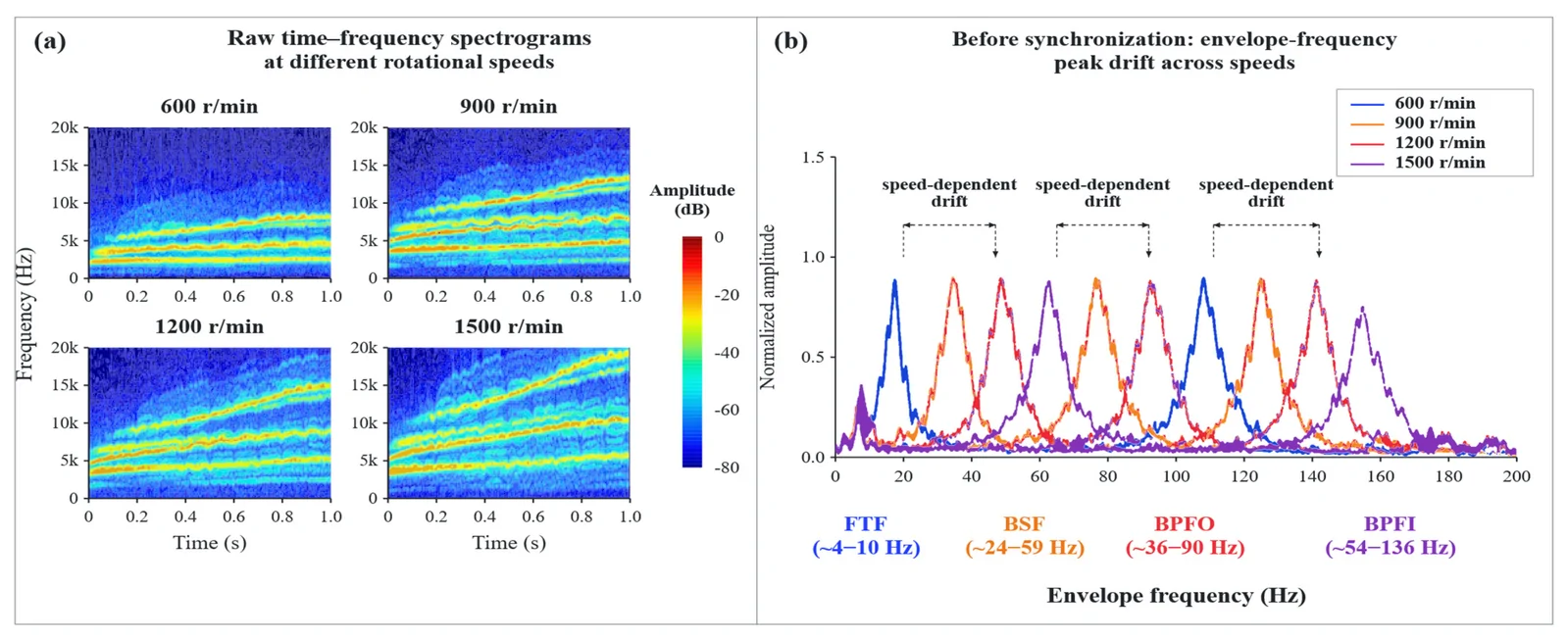
Speed-dependent frequency variation before angular resampling. (**a**) Raw time-frequency spectrograms at rotational speeds of 600, 900, 1200, and 1500 r/min. (**b**) Envelope-frequency peak drift across different speeds, where the curves represent different fault mechanisms obtained from their corresponding fault-class records.

**Figure 9:**
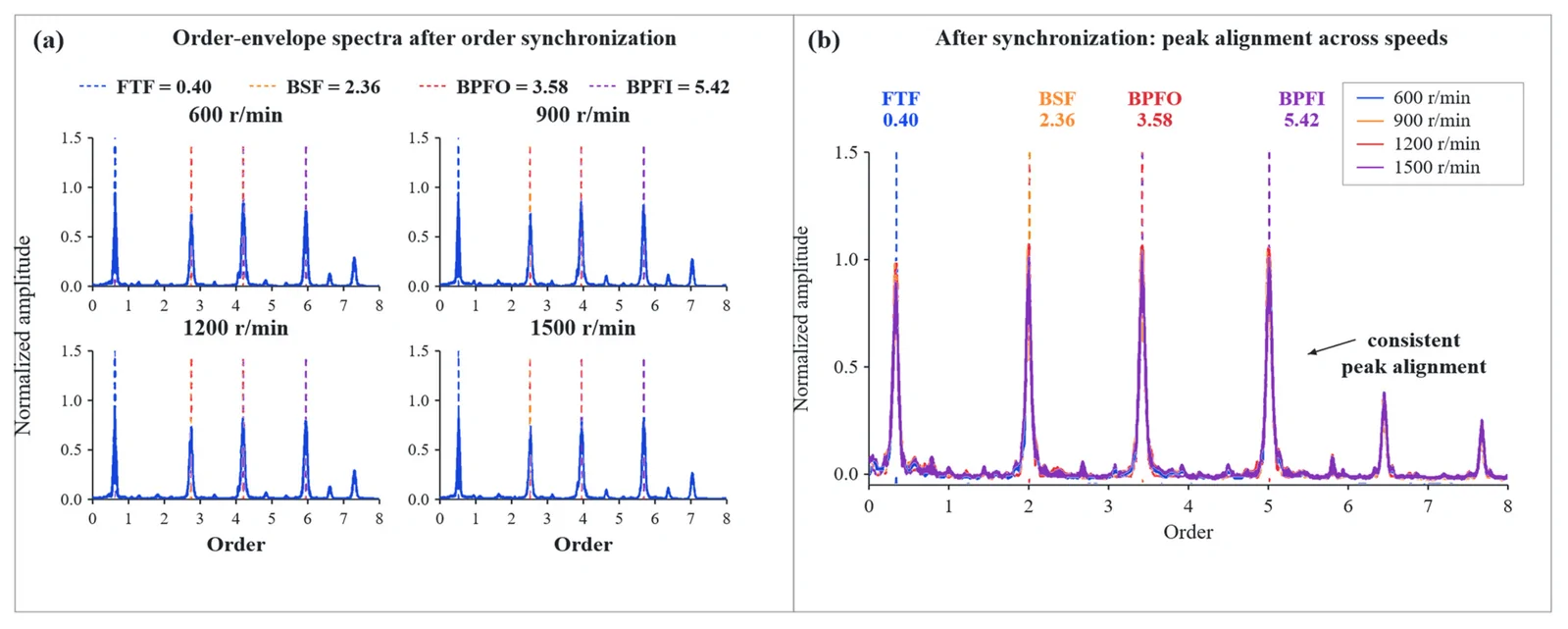
Characteristic-order alignment after angular resampling. (**a**) Order-envelope spectra at four rotational speeds (600–1500 r/min), with dashed lines indicating theoretical fault characteristic orders (FTF, BSF, BPFO, BPFI). (**b**) Overlay of spectra across all speeds, showing consistent peak alignment across fault families.

The two figures (Figure 8 and Figure 9) distinguish speed-dependent frequency displacement from characteristic-order alignment after resampling. FTF-, BSF-, BPFO-, and BPFI-related curves are obtained from cage-fault, rolling-element-fault, outer-race-fault, and inner-race-fault records, respectively. Their joint presentation summarizes different fault classes.

As shown in Table 11, Equal-angle resampling improved the mechanical-scale consistency of acoustic features across speeds. The mean peak-alignment error of the fixed-time representation was 0.31 ± 0.08 order, decreasing to 0.08 ± 0.03 after equal-angle resampling. With PC-LAAPR, the error further decreased to 0.06 ± 0.02 order, an approximately 80.6% reduction relative to the fixed-time representation.

At the same time, the characteristic-band concentration ratio increased from 0.54 to 0.79, and the cosine similarity of envelope-order spectra across speed ranges increased from 0.72 to 0.92. These results show that the improved classification performance was accompanied by lower peak-alignment error and stronger concentration of order-domain energy.

### 3.3. Cross-Load, Cross-Microphone, and Compound Operating-Condition Shifts

The external multi-condition dataset was used to evaluate transfer under load and microphone-domain changes. To prevent leakage of synchronized events, P3 isolates both microphone channels and operating time blocks; its result therefore reflects the combined difficulty of an unseen microphone and unseen operating events. OSAP-KANFormer achieved a Macro-F1 of 95.1 ± 1.0% under leave-one-load-out evaluation and 91.2 ± 1.5% when one microphone was completely excluded from training. Cross-microphone transfer was more difficult because sensitivity, distance, directivity, and the transfer function alter the measured resonance envelope. The bounded order response learned by PC-LAAPR improved robustness to part of this transfer-function variation but did not eliminate the sensor-domain shift.

In Table 10, Under the predefined simultaneous speed-load-microphone shift, OSAP-KANFormer retained a Macro-F1 of 87.0 ± 2.0%, compared with 70.8 ± 2.5% for ASFE-Transformer. The source-record-level confusion matrix remained predominantly diagonal. Under the compound-shift protocol, some cage-fracture records were classified as healthy. Because speed, load, and microphone conditions changed jointly, this comparison does not isolate the contribution of an individual domain factor.

### 3.4. Robustness to Acoustic Disturbances and Speed-Reference Errors

To balance comparability with previous studies and relevance to industrial operation, robustness was evaluated using additive white Gaussian noise (AWGN), recorded factory background noise, and synthetic reverberation. AWGN was applied at SNRs of 0 and −5 dB. The recorded factory noise contained representative fan and gear-meshing backgrounds. Five independent noise realizations were used for every condition.

As shown in Table 12, OSAP-KANFormer achieved Macro-F1 scores of 94.6 ± 0.9% and 89.1 ± 1.3% at 0 and −5 dB AWGN, respectively, and 90.3 ± 1.2% and 89.0 ± 1.4% under recorded factory noise and synthetic reverberation. Relative to ASFE-Transformer, the improvements under the four disturbances were 2.8, 5.5, 5.8, and 6.1 percentage points. The variability of all models increased with disturbance severity, whereas OSAP-KANFormer retained comparatively low standard deviations.

A supplementary sensitivity analysis evaluates errors in the speed reference under the Case 2 constant-speed-to-variable-speed protocol. The trained models, source-record partitions, and acoustic recordings are held fixed, while the speed trajectory supplied to angular integration is perturbed. The comparisons include relative zero-mean speed noise and positive and negative synchronization offsets. Macro-F1 is 90.5% with the unperturbed reference, 88.6% with 2% relative speed noise, and 90.0% and 89.9% with synchronization offsets of +0.5 and −0.5 ms, respectively. These tests characterize sensitivity to the reconstructed speed reference. Missing-pulse recovery is not evaluated; the original quality-screening rule is retained.

### 3.5. Computational Efficiency and Deployment Characteristics

Computational cost is evaluated using model size, FLOPs, batch-one latency, and measured batch throughput. The original parameter and FLOP estimates are retained in Table 13, while runtime is measured on the same hardware using the same physical acoustic observation intervals for all compared methods. Preprocessing includes input preparation, angular resampling where required, and host-to-device transfer. Network inference includes the model-specific analytic frontend, feature encoder, classifier, and probability calibration. For OSAP-KANFormer, PC-LAAPR is included in network inference rather than counted again as preprocessing. End-to-end computational latency is measured independently from the available in-memory acoustic and speed arrays to the returned diagnostic probabilities. Signal acquisition, disk loading, and network communication are outside this timing boundary.

Batch-one latency is reported as the median over repeated synchronized measurements after warm-up. Throughput is measured separately at a batch size of 64 and calculated as the total number of processed samples divided by the elapsed end-to-end processing time. It is not inferred from the reciprocal of batch-one latency. Computational latency is distinguished from observation duration: a four-revolution sample requires 240/n seconds of signal acquisition at a constant speed of n r/min. The streaming update interval additionally depends on overlap and buffering.

The complete pipeline has a median batch-one computational latency of 1.03 ms and a measured batch-64 throughput of 6315 samples/s. Its preprocessing and network-inference times are 0.25 ms and 0.78 ms, respectively. The comparison therefore includes the cost of angular processing and the learned analytic representation. These measurements describe computational processing of available observations and do not include the time needed to acquire a complete diagnostic segment.

### 3.6. Ablation and Sensitivity Analyses

#### 3.6.1. Cumulative Contribution of Individual Modules

To distinguish performance gains from mechanical-scale alignment, adaptive analytic representation, amplitude phase interaction, and concept-evidence classification, the modules were added sequentially to the ASFE-Transformer baseline under identical source-record splits, training epochs, and random seeds. The sequence consisted of OS-AFEM, PC-LAAPR, RAPCA, the physical concept bottleneck, and sparse Concept-KAN. Unlike component-removal ablation, this experiment directly shows the marginal contribution of each added module.

Table 14 reports Macro-F1 and ECE for Case 1 under the strict in-domain P0 protocol, together with Macro-F1 for Case 2 under P1 leave-one-speed-out and P4 constant-speed-to-variable-speed transfer. ECE is reported only for the fixed-condition cumulative models. OS-AFEM improved fixed-condition performance by only 0.4 percentage points but produced substantially larger gains under variable-speed evaluation, indicating that its primary role is to remove the mechanical-scale mismatch of fixed time frames. Adding PC-LAAPR further improved the two variable-speed metrics, showing that a smooth complex residual initialized by the Hilbert transform can adapt to order bands affected by microphone response and acoustic transmission paths.

RAPCA further improved leave-one-speed and variable-trajectory F1, supporting the use of phase coherence to screen amplitude transients with consistent periodicity. Introducing the concept bottleneck did not produce a clear classification gain and slightly reduced leave-one-speed F1, reflecting the information constraint imposed by forcing the decision through a low-dimensional physical-concept space. Under Case 2/P1, Concept-KAN increased Macro-F1 from 91.3% to 92.4%. Under Case 1/P0, it reduced ECE from 0.030 to 0.026. Its main value is therefore to organize concept evidence through sparse nonlinear functions rather than to gain performance simply by increasing classifier capacity.

The additional ablation separates the effects of the auxiliary training objective and the concept-level classifier. In the no-auxiliary-loss variant, the auxiliary-loss weight is set to zero from the beginning of Stage 1 and remains zero throughout training. The main classification loss, concept supervision, remaining regularizers, data partitions, training seeds, and validation-based checkpoint selection remain unchanged. The concept-MLP comparison retains the physical-concept predictor and replaces only the concept-to-class classifier. In-domain results refer to Case 1/P0, while leave-one-speed-out performance, concept-prediction quality, and explanation stability refer to Case 2/P1.

Beyond the mean correlation reported in Table 15, 120 common source-record–fold units were eligible for analysis, each evaluated using the ten seed pairs formed by five training seeds, yielding 1200 correlation coefficients per model. The median Spearman correlation was 0.86 for the complete OSAP-KANFormer, with an interquartile range of 0.81–0.90 and a 5th–95th percentile interval of 0.68–0.96. The corresponding values were 0.79, 0.71–0.85, and 0.52–0.94 without the auxiliary classification loss, and 0.72, 0.59–0.81, and 0.38–0.93 for the concept MLP. No undefined coefficients were present. This pattern indicates higher central ranking agreement and a narrower middle range for the complete model, while the overlapping percentile intervals show that its advantage is not uniform across all records and seed pairs. These intervals describe the empirical spread of correlation coefficients. Because the coefficients shared source records and trained models, they were treated as dependent descriptive observations.

Under the reported configuration, removing the auxiliary classification loss reduced leave-one-speed-out Macro-F1 from 92.4% to 85.7%, compared with a reduction from 97.3% to 96.2% under the in-domain protocol. This result indicates that the training objective materially affects cross-speed performance in the evaluated setting. It does not establish that the auxiliary objective preserves complete concept semantics. Replacing Concept-KAN with a concept MLP reduced leave-one-speed-out Macro-F1 by 1.1 percentage points, while the reported contribution-ranking correlation decreased from 0.84 to 0.70. The classification difference and the reproducibility of explanations are therefore interpreted separately.

#### 3.6.2. Sensitivity Analysis

One-factor-at-a-time sensitivity experiments were conducted under the Case 2/P1 leave-one-speed-out protocol. The varied factors were the structural parameters ($N_{r},k,L_{\theta },Q$) and training weights ($\lambda_{c},\lambda_{sp}$). All candidate ranges were specified a priori with five independent random seeds, and hyperparameters were selected using only training and validation splits. A range was considered stable when Macro-F1 remained within 0.6 percentage points of the best value with overlapping uncertainty intervals.

**Figure 10:**
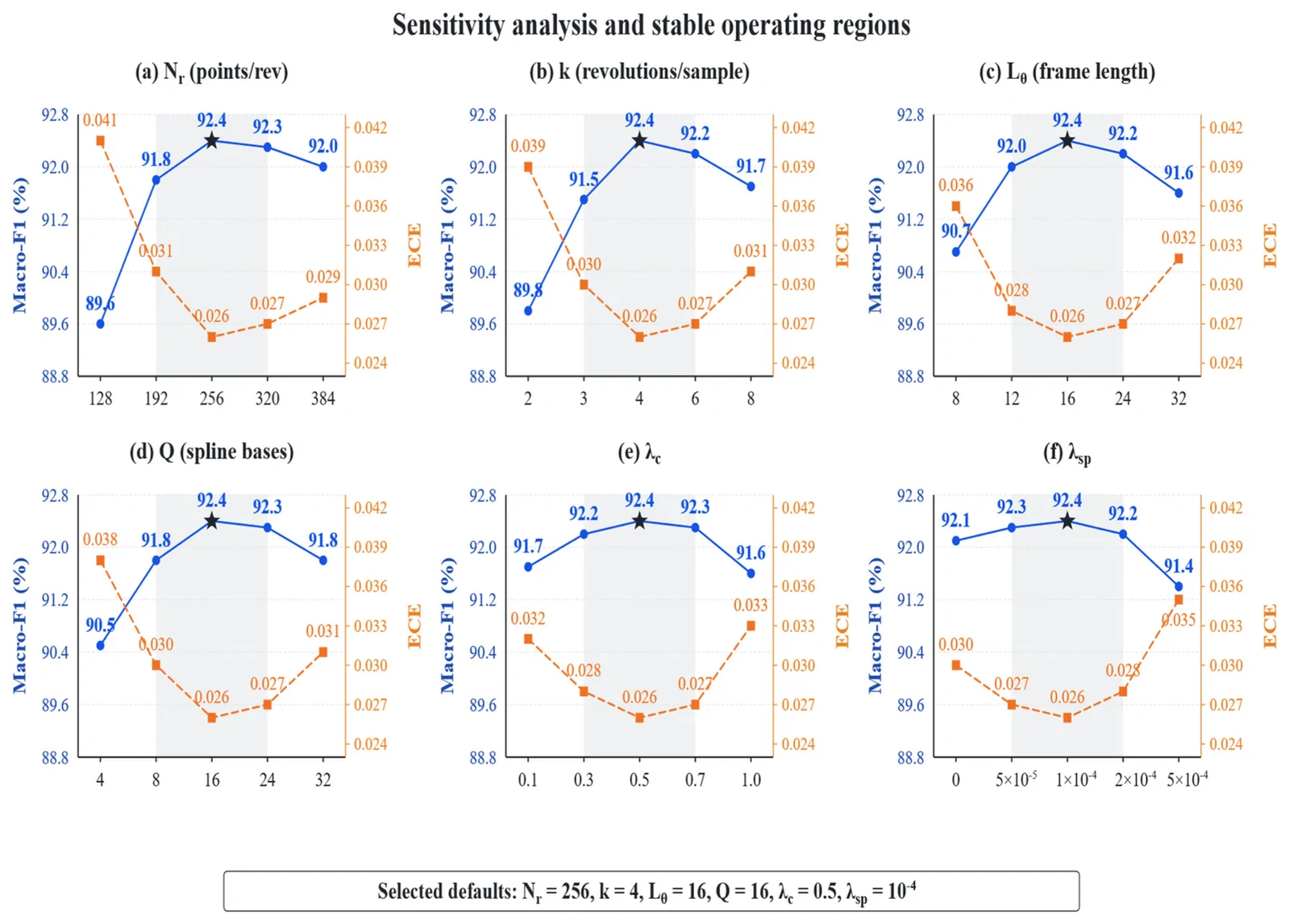
Sensitivity analysis and stable operating regions.

For the structural parameters, Figure 10 shows that none produced an isolated, sharp optimum. Increasing $N_{r}\;$ from 128 to 256 improved Macro-F1 from 89.6% to 92.4%, after which 192–320 formed a flat region with marginal gains but increased interpolation cost. Similarly, performance saturated at k=4–6, $L_{\theta }=12–24$, and Q=16–24. Outside these ranges, too short a window or too few spline bases failed to capture the complete impact morphology, while overly long windows reduced local angular resolution. Defaults of $N_{r}=256$, k=4, $L_{\theta }=16$, and Q=16 were selected within these stable, efficiency-performance regions.

For the training weights, moderate concept supervision was beneficial: increasing $\lambda_{c}$ from 0.1 to 0.5 raised mean concept $R^{2}$ from 0.821 to 0.891 and Macro-F1 by 0.7 percentage points, whereas excessive constraints ($\lambda_{c}=1.0$) suppressed discriminative information and reduced Macro-F1. The stable range was $\lambda_{c}=0.3–0.7$. Regarding sparsity, without group sparsity the model retained 55 active concept-to-class connections with an explanation stability of 0.73. Setting $\lambda_{sp}=1\times 10^{-4}$ reduced the connections to 24 and increased stability to 0.84 without a Macro-F1 drop, while excessive sparsity ($5\times 10^{-4}$) removed useful mechanical evidence. Therefore, $\lambda_{c}=0.5$ and $\lambda_{sp}=1\times 10^{-4}$ were adopted. Overall, the selected defaults lie in flat regions rather than at search boundaries, indicating the main conclusions are robust to single-parameter variations.

Pruning-threshold sensitivity is evaluated using thresholds of 0, 0.01, 0.025, 0.05, 0.075, and 0.10 while keeping the group-sparsity weight fixed. For each random seed, all threshold variants start from the same Stage 2 checkpoint and use the same fine-tuning budget. Threshold selection is based on validation Macro-F1.

As shown in Table 16, The pruning threshold is selected using validation performance, balancing accuracy and sparsity. At the selected threshold of 0.05, 24 concept-to-class connections remain, and the corresponding test Macro-F1 is 92.4%. Increasing the threshold to 0.10 reduces test Macro-F1 to 90.7%, indicating a loss of useful evidence under stronger pruning. The result characterizes the accuracy–sparsity trade-off within the tested configurations rather than establishing a universally optimal threshold.

Training–validation behavior was examined using the saved learning curves for Case 2/P1. Checkpoints were selected using validation Macro-F1 before test evaluation. At the selected checkpoints, mean training, validation, and test Macro-F1 values were 96.1%, 93.4%, and 92.4%, respectively. The remaining training–validation gap is reported rather than interpreted as absent, and P1’s reuse of bearing entities across different speeds is retained as a limitation of the generalization assessment.

### 3.7. Mechanistic and Interpretability Analyses

**Figure 11:**
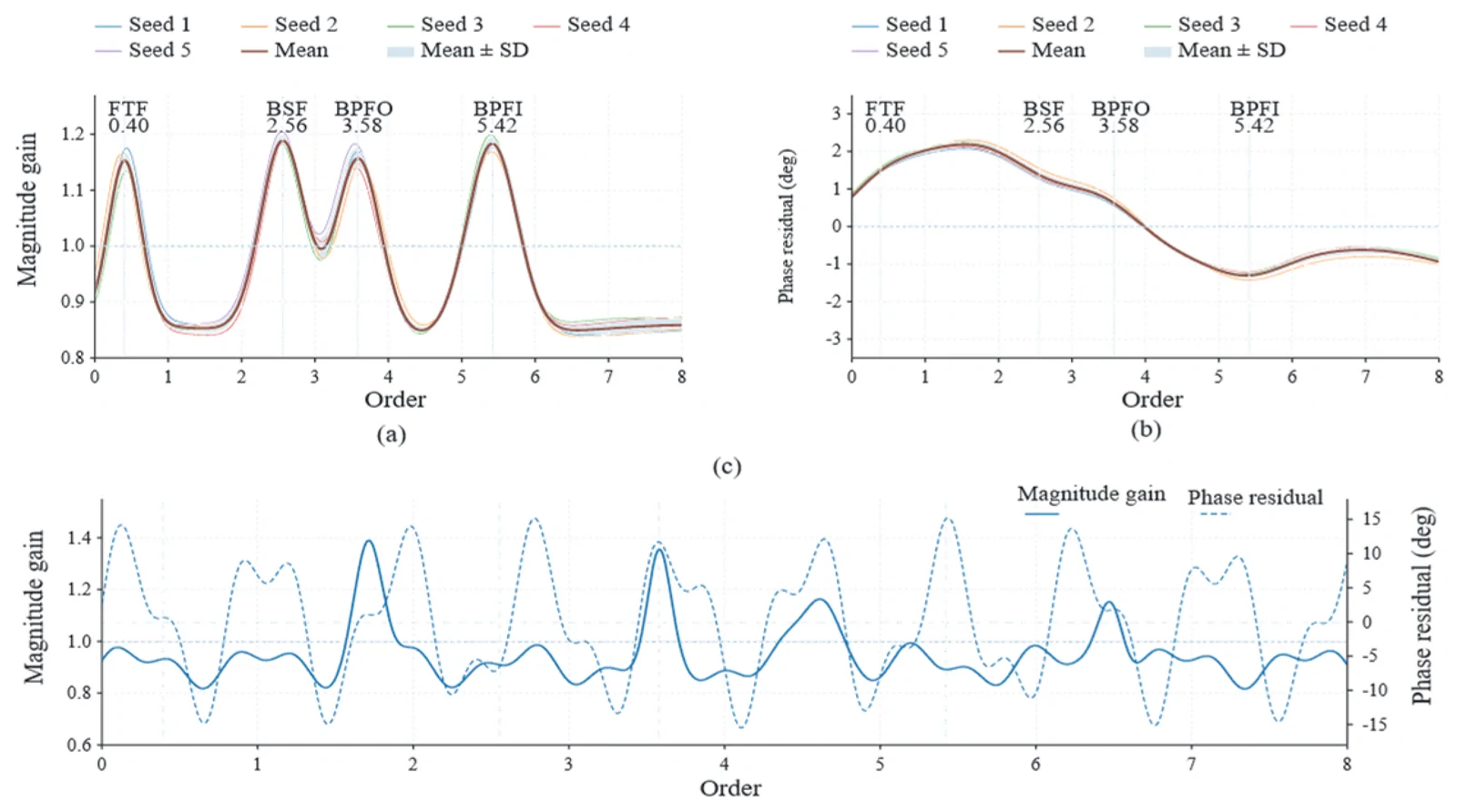
PC-LAAPR amplitude and phase responses across seeds and a representative response of the unconstrained complex filter. (**a**) PC-LAAPR magnitude response across five seeds. (**b**) Smooth and bounded phase residual. (**c**) Representative response of an unconstrained complex filter.

Figure 11 presents the amplitude responses and phase residuals learned by PC-LAAPR across five random seeds. The amplitude responses exhibit broad and smooth enhancement near BPFI-, BPFO-, BSF-, and FTF-related order bands. Curves from different seeds remain similar, with a limited standard-deviation band around the mean. The phase residual changes smoothly around zero and remains within ±3.4°, without the high-frequency alternating oscillation observed for the unconstrained complex filter. These results indicate that the explicit analytic-structure constraint improves response repeatability.

The learned responses in Figure 11 exhibit visible seed-to-seed differences, and the enhancement is not identical at every theoretical fault order. Some order intervals remain close to unit gain, indicating that the network selectively adjusts the response according to the acoustic evidence in the training data. To illustrate a possible non-ideal learned response, Figure 11c shows an unconstrained complex filter from the seed with median validation Macro-F1. This response contains narrow local over-enhancement and stronger fluctuations outside the target order regions. Although its characteristic-band SNR reaches 8.8 dB, its cross-position Macro-F1, cross-seed response correlation, and out-of-band stability are all lower than those of PC-LAAPR.

Table 17 summarizes the prediction quality of the eleven physical concepts. The mean normalized MAE, mean R^2^, and mean Pearson correlation are 0.070, 0.891, and 0.931, respectively. BPFI-order energy has the highest reported R^2^ of 0.94, whereas phase-increment dispersion has the lowest value of 0.84. These results characterize agreement between predicted concepts and the specified physical targets under the evaluated protocol. They do not establish completeness of the concept set, absence of unintended information encoding, or the sign of each concept’s contribution to a particular class.

**Figure 12:**
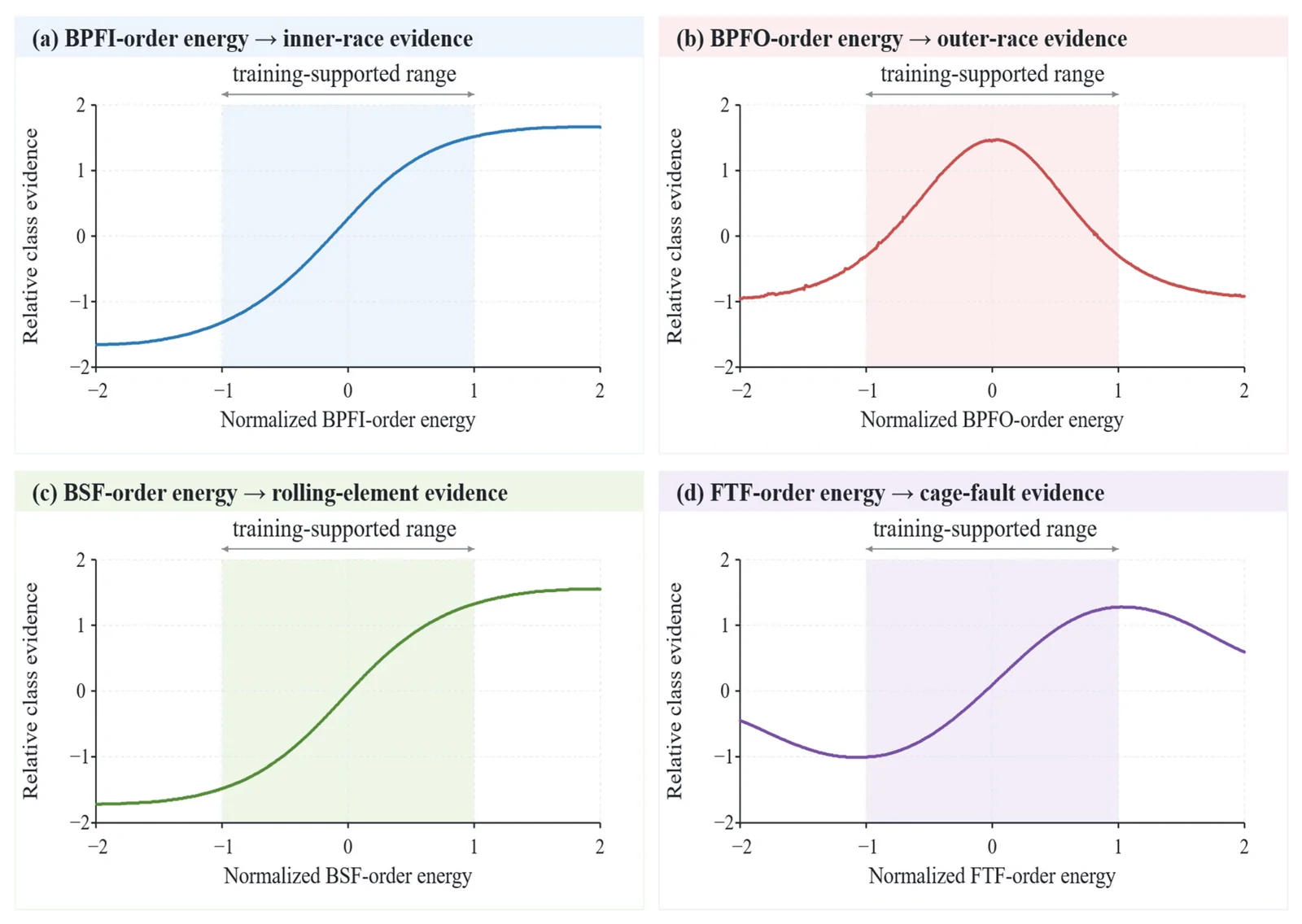
Learned concept-to-class evidence functions.

Figure 12 presents the concept-to-class functions extracted from the trained Concept-KAN classifier. The four panels correspond to BPFI-order energy and the inner-race class, BPFO-order energy and the outer-race class, BSF-order energy and the rolling-element class, and FTF-order energy and the cage-fault class, respectively. The complete edge function, including its linear and spline components, is evaluated over the concept range observed in the training partition, with shaded regions indicating the training-supported range. Contributions are expressed relative to the corresponding healthy-training median.

The learned curves exhibit distinct evidence-mapping behaviors: BPFI-order energy and BSF-order energy show monotonically increasing evidence within the training-supported range, confirming their positive correlation with inner-race and rolling-element faults, respectively. In contrast, BPFO-order energy exhibits a peak-shaped response, with maximum evidence centered within the supported range, while FTF-order energy shows a non-monotonic valley-to-rise pattern. These behaviors indicate that the soft monotonicity prior guides, but does not rigidly enforce, the functional shape, allowing the model to capture nonlinear and non-monotonic relationships where mechanically justified.

### 3.8. Stratified Error Analysis and a Representative Failure Case

Held-out predictions from Case 2/P1 are summarized separately by test speed, sensor position, and fault category. For each subgroup, the error rate is calculated as the proportion of incorrectly classified test windows within each seed and then averaged across seeds. The error rates across the four held-out speeds range from 5.8% to 9.2%, while the axial-side and end-side rates are 6.2% and 8.4%, respectively. Class-wise confusion patterns are shown in Figure 7 in Section 3.2. These stratified summaries describe the distribution of errors within the evaluated test population; they do not isolate causal effects because speed, sensor position, and fault category may be associated. The separate trajectory example below illustrates one concept-level failure mechanism.

A separate Case 2 trajectory record is examined to illustrate a representative failure. The true condition is cage fault, and the record follows a 600 → 1500 → 600 r/min compound speed sweep. The model assigns probabilities of 0.46 to rolling-element fault and 0.39 to cage fault, giving a top-two margin of 0.07. The FTF-order energy is 0.48, below the median value of 0.72 for correctly classified cage-fault training samples, whereas the BSF-order energy and sideband ratio are 0.61 and 0.67. These values indicate competing concept evidence.

The record was acquired during low main-drive torque and a rapid speed sweep. However, radial bearing load was not measured directly, so the weak FTF-related component cannot be attributed solely to bearing load. Operating conditions, structural transmission, and speed transients provide possible contributing explanations, but their individual effects are not isolated by this record.

**Figure 13:**
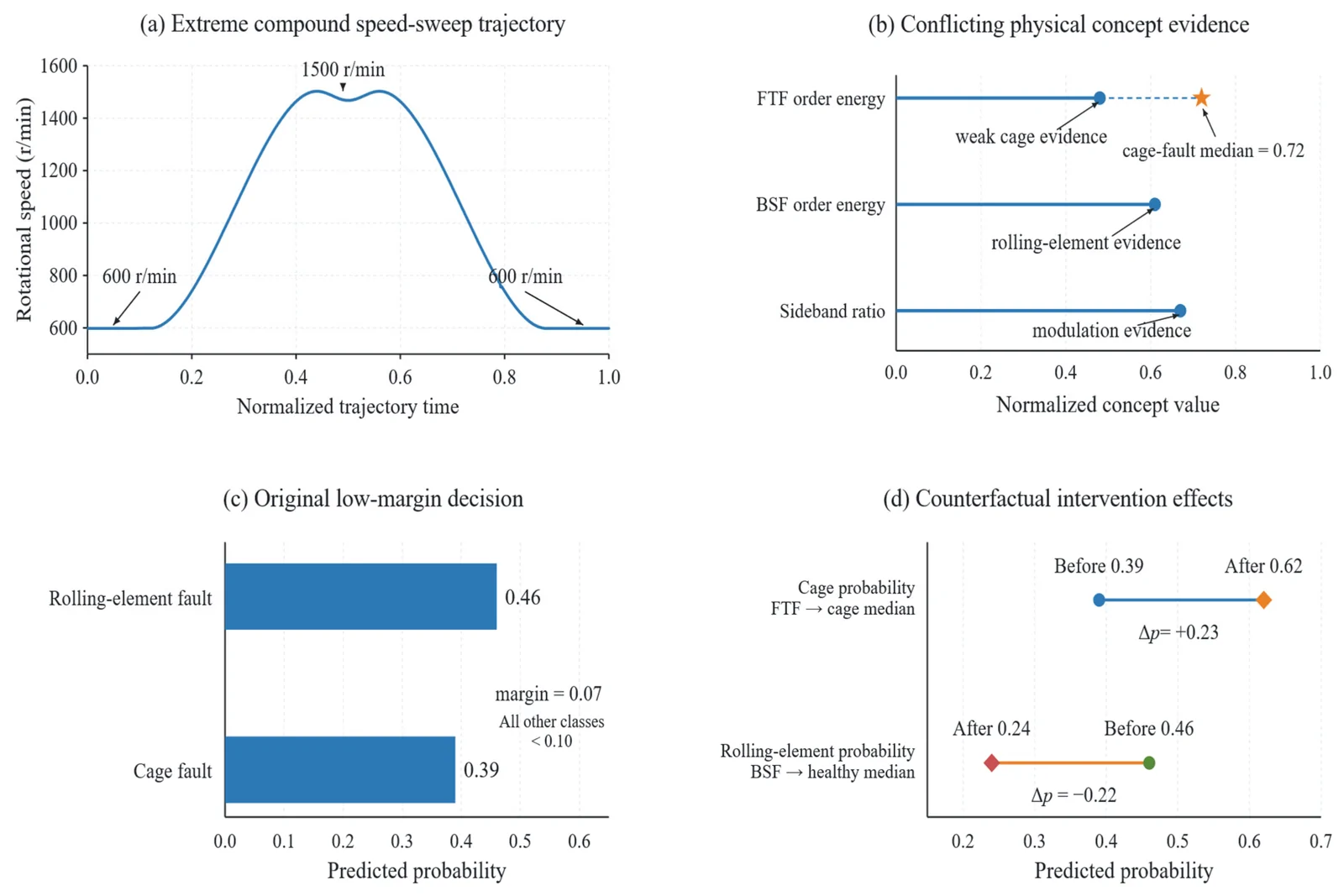
Physical-concept evidence and counterfactual analysis of a representative misclassified sample under an extreme compound speed sweep.

Two separate concept interventions illustrate the model’s decision dependence. Replacing FTF-order energy with the cage-fault training median increases the cage-fault probability from 0.39 to 0.62. Replacing BSF-order energy with the healthy-range training median reduces the rolling-element-fault probability from 0.46 to 0.24. Replacement values are obtained from the training data and restricted to the corresponding 1st–99th percentile ranges. These changes characterize concept dependence within the trained model. Figure 13 shows Physical-concept evidence and counterfactual analysis of a representative misclassified sample under an extreme compound speed sweep.

### 3.9. Limitations and Scope of Application

**Figure 14:**
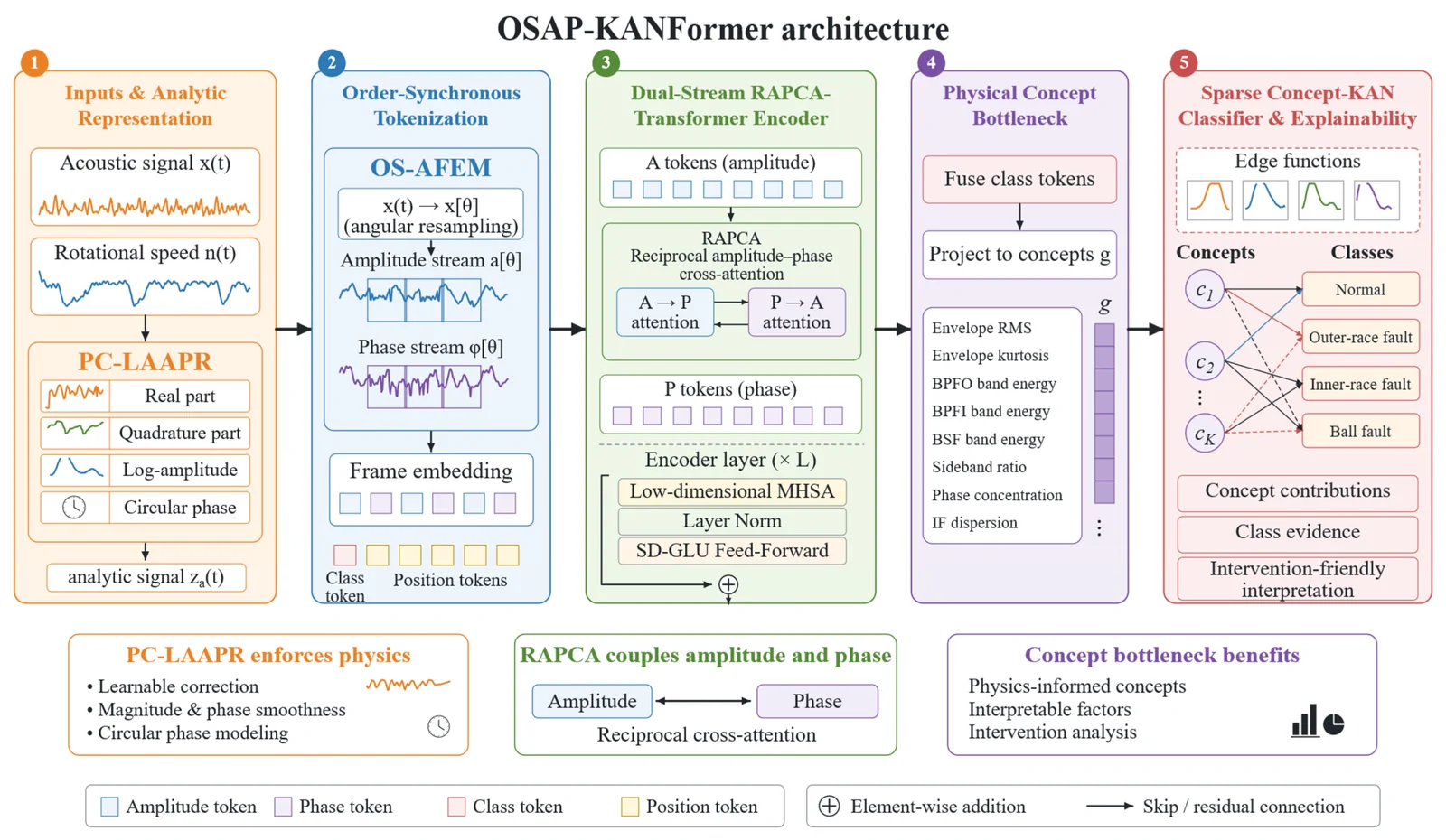
Core concept of the proposed method.

Figure 14 shows the Core concept of the proposed method. OSAP-KANFormer requires a speed reference that supports a valid angular mapping. Limited measurement disturbances and invalid speed records are different operating situations: the former may degrade classification, whereas the latter may prevent a diagnostic output. Consequently, classification performance should be considered together with diagnostic coverage when corrupted inputs are rejected. Equal-angle resampling also does not remove sensor-transfer differences or load-dependent structural transmission.

The physical-concept set is designed for the evaluated bearing configurations and fault categories. Geometry-dependent order energies and the associated sideband descriptor cannot be assumed to remain valid for unknown bearing geometry, distributed damage, or compound faults. The evaluated results therefore apply to the specified concept configuration. Performance with a reduced geometry-independent concept set has not been evaluated, and no claim is made that the existing model can be directly applied to unknown bearing geometry or unobserved compound-fault categories.

The absence of a latent-feature bypass at inference is an architectural property. Joint classification training can influence continuous concept representation. Concept-prediction errors, comparable contribution rankings, and intervention tests therefore provide complementary evidence about the decision pathway. These analyses concern the trained model’s internal dependence on concepts and do not establish physical causality. Practical benefits to operators require a separate domain-expert study.

The three datasets are trained independently. Their results support evaluation of the framework across several sensing configurations but do not demonstrate zero-shot transfer of a single set of model weights across datasets. Similarly, P1 evaluates unseen speeds for previously observed bearing entities and must be distinguished from entity-disjoint evaluation.

Relationships to fault-tolerant operation, fault-class probabilities and concept-level evidence may provide condition information to a supervisory monitoring system. They do not, by themselves, establish whether control reconfiguration is feasible or sufficient. Control-structure selection considers accessibility and observability of the available sensing and actuation configuration [27], whereas robust fault-tolerant predictive control addresses closed-loop behavior under uncertainty and faults [28]. The present study evaluates diagnostic information. Integration into a control loop would additionally require severity estimation, uncertainty propagation, operating constraints, and closed-loop validation.

## 4. Conclusions

This study presented OSAP-KANFormer for bearing fault diagnosis using synchronized acoustic and rotational-speed measurements. Equal-angle resampling establishes a common mechanical scale across operating speeds, while constrained amplitude–phase learning and reciprocal cross-attention support the representation of acoustic transients. A physical-concept bottleneck and an additive KAN provide an explicit concept-to-class decision pathway.

The reported results indicate that the main performance benefit occurs under changing speed conditions rather than on the fixed-speed benchmark. Macro-F1 reached 92.4% in leave-one-speed-out evaluation and 90.5% in constant-speed-to-variable-speed transfer, exceeding ASFE-Transformer by 7.3 and 9.2 percentage points, respectively. Compared with OS-ASFE, the corresponding improvements were 2.7 and 2.0 percentage points. These comparisons distinguish the contribution of mechanical-angle alignment from the additional benefit of the subsequent model components.

Concept-KAN provided a smaller classification improvement over a concept MLP, with its principal architectural benefit being the explicit representation of concept-to-class evidence. The interpretation of this evidence remains subject to concept-prediction error, training-path effects, and the coverage of the selected concept set. P1 concerns unseen speeds for previously observed bearing entities, and separately trained dataset evaluations should not be interpreted as cross-dataset deployment without re-training.

Future work will investigate uncertainty-aware angular mapping, reduced-geometry and compound-fault representations, and transfer across independently acquired datasets. Integrating the diagnostic outputs into maintenance or control decisions will require further validation beyond classification performance.

## Figures and Tables

**Table 1:** Physical concepts used in the concept bottleneck.

No.	Concept	Physical Meaning	Definition
c1	Envelope RMS	Overall energy of the demodulated acoustic signal	$c_{1}=RMS(a)=\sqrt{\frac{1}{N}\sum_{i=1}^{N}a_{i}^{2}}$
c2	Envelope kurtosis	Non-Gaussian impulsiveness	$c_{2}=\frac{\frac{1}{N}\sum_{i=1}^{N}(a_{i}-\mu_{a}{)}^{4}}{\sigma_{a}^{4}+\varepsilon }$
c3	Crest factor	Ratio of peak amplitude to background level	$c_{3}=\frac{\underset{i}{max}|a_{i}|}{RMS(a)+\varepsilon }$
c4	Order-spectrum entropy	Dispersion of energy in the order domain	$c_{4}=-\frac{\sum_{q=1}^{Q}p_{q}logp_{q}}{\log Q}$
c5	BPFI-order energy	Evidence of an inner-race fault	$c_{5}=\sum_{h=1}^{H}B(h\;o_{BPFI})$
c6	BPFO-order energy	Evidence of an outer-race fault	$c_{6}=\sum_{h=1}^{H}B(h\;o_{BPFO})$
c7	BSF-order energy	Evidence of a rolling-element fault	$c_{7}=\sum_{h=1}^{H}B(h\;o_{BsF})$
c8	FTF-order energy	Evidence related to cage motion or trajectory	$c_{8}=\sum_{h=1}^{H}B(h\;o_{FTF})$
c9	Sideband ratio	Amplitude modulation around fault orders	$c_{9}=\frac{E_{sb}}{E_{ctr}+\varepsilon }$
c10	Phase concentration	Circular consistency of phase increments	$c_{10}=|\frac{1}{N}\sum_{i=1}^{N}exp\left(j\Delta \phi_{i}\right)|$
c11	Phase-increment dispersion	Local fluctuation of instantaneous order	$c_{11}=\frac{\mathrm{MAD}\left(\Delta \phi \right)}{median\left(|\Delta \phi |\right)+\varepsilon }$

**Table 2:** Overview of the datasets and their roles in evaluation.

Dataset	Health Conditions	Speed	Load	Acoustic Acquisition	Sampling Rate	Evaluation Role
Case 1: ASFE benchmark	Healthy and nine local fault conditions	900 r/min	5 kN	Single acoustic channel	20 kHz	Reproduction and split-sensitivity analysis
Case 2: variable-speed cold-rolling field dataset	Healthy, inner-race, outer-race, rolling-element, and cage faults	Four speed ranges; linear and nonlinear acceleration/deceleration trajectories	PLC torque proxy; low/medium/high groups defined by training-set tertiles	Two magnetic multimodal sensors plus a 1024-pulse/rev encoder	51.2 kHz; acoustic, speed, and PLC variables synchronized	P1/P4 variable-speed validation
Case 3: MCAB-CF	Healthy and cage fracture	800/1000/1200 r/min	0/15/30% of rated torque	Three microphones	32.768 kHz	Cross-speed, load, microphone, and compound-shift evaluation

**Table 3:** Composition of the cold-rolling field dataset.

Class	Independent Bearings/Events	Source Records	Production Batches	Training	Validation	Testing
Healthy	12	72	36	48	12	12
Inner-race fault	5	29	15	17	6	6
Outer-race fault	5	28	15	16	6	6
Rolling-element fault	5	23	12	11	6	6
Cage fault	5	23	13	11	6	6
Total	32	175	91	103	36	36

**Table 4:** Cross-condition evaluation protocols.

ID	Name	Split Strategy	Purpose	Related Section	Dataset
P0	Strict in-domain	Non-overlapping source records under matched conditions	Leakage-free baseline classification	Section 3.1 and Section 3.6.1	Case 1
P1	Leave-one-speed-out	All but one speed are used for training; testing is performed at the unseen speed	Unseen-speed generalization for previously observed bearing entities	Section 3.2, Section 3.6.1, Section 3.7 and Section 3.8	Case 2
P2	Leave-one-load-out	All but one load level are used for training	Load robustness	Section 3.3	Case 3
P3	Leave-one-microphone-out	Two microphones are used for training and the third for testing	Sensor/position robustness	Section 3.3	Case 3
P4	(1) Constant-speed to variable-speed trajectory; (2) leave-one-trajectory-out	Constant-speed or partial-trajectory data are used for training and unseen variable-speed trajectories for testing	Non-stationary speed extrapolation	Section 3.2, Section 3.6.2, Section 3.7 and Section 3.8	Case 2
P5	Compound shift	Training and validation use 800/1000 r/min, 0/15% load, and microphones M1/M2; testing uses only 1200 r/min, 30% load, and microphone M3	Simultaneous generalization to unseen speed, load, and microphone	Section 3.3 and Section 3.8	Case 3
P6	Acoustic disturbance	AWGN, recorded factory background noise, and synthetic reverberation	Environmental robustness	Section 3.4	Case 11

**Table 5:** Baseline and ablation-oriented comparison methods.

Method	Comparison Role
MA1DCNN	Lightweight convolutional acoustic baseline
Transformer	Standard waveform/frame Transformer
LiConvFormer	Lightweight convolutional Transformer [8]
ASFE-Transformer	Reproduced analytic-frame baseline [10]
VSF-ST	Speed-fusion Transformer for variable-speed diagnosis [18]
LWPI	Learnable wavelet-driven physically interpretable network adapted to acoustic signals [16]
ASFE+ hidden-feature KAN	KAN replaces the linear classifier; no concept bottleneck
OS-ASFE	ASFE with equal-angle resampling only
PC-LAAPR + ASFE	Learnable analytic representation without RAPCA/Concept-KAN
OSAP-KANFormer	Complete proposed method
BEATs	Task adaptation of a pre-trained audio model [24]
SSAMBA	Self-supervised state-space audio baseline [25]

**Table 6:** Default implementation and hyperparameter settings.

Item	Setting
Angle-domain resampling	256 points/revolution; 4 revolutions/sample; 1024 points in total
OS-AFEM	Frame length 16; hop size 8; 127 frame tokens + class token
Embedding/encoder	D = 64; 4 layers; 4 heads; SD-GLU expansion ratio 2; convolution kernel 5
PC-LAAPR	16 cubic B-spline bases; Hilbert-residual initialization
RAPCA	One reciprocal cross-attention module; per-head dimension 8
Concept-KAN	11 concepts; cubic splines; 8 initial grid intervals; group sparsity
Optimization	Adam; initial learning rate 1 × 10^−3^; weight decay 1 × 10^−4^; cosine decay; 5-epoch warm-up
Training	Batch size 64; at most 80 epochs; early stopping patience 12
Repeated trials	Five independent random seeds
Software/hardware	PyTorch 2.5.1; CUDA 12.4; NVIDIA GeForce RTX 5090 24 GB; Intel Core i9-14900K CPU; 64 GB RAM

**Table 7:** Fixed-condition results under the leakage-free source-record protocol.

Model	Accuracy (%)	Macro-F1 (%)	ECE	Parameters (M)
MA1DCNN	95.1 ± 0.8	94.7 ± 0.9	0.061	0.07
Transformer	95.8 ± 0.7	95.4 ± 0.8	0.054	0.42
LiConvFormer	96.5 ± 0.5	96.2 ± 0.6	0.041	0.32
ASFE-Transformer	96.8 ± 0.6	96.5 ± 0.7	0.038	0.09
OS-ASFE	97.2 ± 0.5	96.9 ± 0.6	0.035	0.18
OSAP-KANFormer	98.0 ± 0.4	97.3 ± 0.5	0.026	0.31

**Table 8:** Acoustic characteristics under different sensor positions and operating stages.

Condition	Characteristic-Band SNR/dB	Fault-to-Healthy Energy Contrast/dB	Cross-Speed Spectral Similarity
Axial-side sensor	9.4 ± 1.2	6.8 ± 1.0	0.91 ± 0.04
End-side sensor	7.2 ± 1.4	5.1 ± 1.1	0.86 ± 0.05
Start-up/acceleration	7.8 ± 1.5	5.4 ± 1.2	0.87 ± 0.06
Steady rolling	10.2 ± 1.1	7.1 ± 0.9	0.93 ± 0.03
Speed adjustment	8.6 ± 1.3	6.0 ± 1.0	0.89 ± 0.05
Deceleration/shutdown	8.0 ± 1.4	5.6 ± 1.1	0.88 ± 0.05

**Table 9:** Generalization across speeds and speed trajectories (%).

Model	Leave-One-Speed-Out Macro-F1	Constant-Speed → Variable-Speed Trajectory	Leave-One-Variable-Speed-Trajectory-Out	Worst-Trajectory F1
ASFE-Transformer	85.1 ± 1.8	81.3 ± 2.1	79.6 ± 2.3	75.8 ± 2.5
VSF-ST	87.6 ± 1.7	83.9 ± 2.0	82.1 ± 2.2	78.4 ± 2.4
LWPI	88.3 ± 1.6	85.1 ± 1.9	83.6 ± 2.1	80.2 ± 2.3
OS-ASFE	89.7 ± 1.5	88.5 ± 1.8	85.0 ± 2.0	81.7 ± 2.2
BEATs	92.6 ± 1.3	89.9 ± 1.6	87.2 ± 2.3	82.3 ± 1.2
SSAMBA	91.9 ± 1.4	90.1 ± 1.6	89.2 ± 2.0	84.6 ± 1.8
OSAP-KANFormer	92.4 ± 1.2	90.5 ± 1.5	89.2 ± 1.7	85.4 ± 2.0

**Table 10:** Cross-condition Macro-F1 on the external acoustic dataset (%).

Model	Leave-One-Load-Out	Leave-One-Sensor-Out	Speed + Load Shift	Compound Shift
ASFE-Transformer	90.2 ± 1.4	82.4 ± 2.0	78.6 ± 2.3	70.8 ± 2.5
OS-ASFE	92.5 ± 1.2	86.7 ± 1.8	83.5 ± 2.1	78.9 ± 2.3
PC-LAAPR + ASFE	93.7 ± 1.1	89.1 ± 1.7	86.2 ± 1.9	82.6 ± 2.2
OSAP without Concept-KAN	94.0 ± 1.1	89.4 ± 1.6	87.0 ± 1.9	84.1 ± 2.1
OSAP-KANFormer	95.1 ± 1.0	91.2 ± 1.5	89.3 ± 1.8	87.0 ± 2.0

**Table 11:** Acoustic spectral alignment before and after order-synchronous processing.

Representation	Peak-Alignment Error/Order	Characteristic-Band Concentration Ratio	Cross-Speed Spectral Cosine Similarity	Characteristic-Band SNR/dB
Fixed-time ASFE representation	0.31 ± 0.08	0.54 ± 0.06	0.72 ± 0.05	5.8 ± 1.1
Equal-angle resampling	0.08 ± 0.03	0.73 ± 0.05	0.89 ± 0.04	8.1 ± 1.0
Equal-angle resampling + PC-LAAPR analytic output	0.06 ± 0.02	0.79 ± 0.04	0.92 ± 0.03	9.4 ± 0.9

**Table 12:** Robustness under additive and structured acoustic disturbances (Macro-F1, %).

Model	Clean	0 dB AWGN	−5 dB AWGN	Factory Noise	Reverberation
ASFE-Transformer	96.5 ± 0.7	91.8 ± 1.2	83.6 ± 1.8	84.5 ± 1.7	82.9 ± 1.9
LiConvFormer	96.2 ± 0.6	92.5 ± 1.1	85.1 ± 1.6	86.2 ± 1.5	84.7 ± 1.7
LWPI	97.0 ± 0.6	93.4 ± 1.0	86.8 ± 1.5	87.5 ± 1.4	86.0 ± 1.6
OSAP-KANFormer	97.3 ± 0.5	94.6 ± 0.9	89.1 ± 1.3	90.3 ± 1.2	89.0 ± 1.4

**Table 13:** Model complexity, computational latency, and measured throughput.

Model	Parameters (M)	FLOPs (M)	Preprocessing Time (ms)	Network Inference Time (ms)	End-to-End Latency (ms)	Throughput, Batch 64 (Samples/s)
ASFE-Transformer	0.09	13.66	0.15	0.45	0.60	10,667
LiConvFormer	0.32	14.40	0.16	0.58	0.74	8769
OS-ASFE	0.10	21.5	0.22	0.67	0.89	7391
OSAP-KANFormer	0.31	26.8	0.25	0.78	1.03	6315

**Table 14:** Cumulative module contributions under different datasets and protocols.

Cumulative Model	Strict In-Domain F1/% (Case 1, P0)	Leave-One-Speed F1/% (Case 2, P1)	Constant-Speed → Variable-Speed Trajectory F1/% (Case 2, P4)	ECE (Case 1, P0)
ASFE-Transformer	96.5 ± 0.7	85.1 ± 1.8	81.3 ± 2.1	0.038
+OS-AFEM	96.9 ± 0.6	89.7 ± 1.5	88.5 ± 1.8	0.035
+PC-LAAPR	97.0 ± 0.6	90.6 ± 1.4	88.7 ± 1.7	0.032
+RAPCA	97.2 ± 0.5	91.7 ± 1.3	89.6 ± 1.6	0.029
+Concept bottleneck and MLP classifier	97.0 ± 0.6	91.3 ± 1.4	89.8 ± 1.6	0.030
+Sparse Concept-KAN (complete model)	97.3 ± 0.5	92.4 ± 1.2	90.5 ± 1.5	0.026

**Table 15:** Ablation results for performance and explanation quality.

Variant	In-Domain F1	Leave-One-Speed F1	Concept R^2^	Cross-Seed Ranking Correlation
Complete OSAP-KANFormer	97.3 ± 0.5	92.4 ± 1.2	0.90 ± 0.02	0.84 ± 0.03
Without auxiliary classification loss	96.2 ± 0.8	85.7 ± 1.8	0.87 ± 0.04	0.78 ± 0.04
Concept MLP classifier	97.0 ± 0.6	91.3 ± 1.4	0.88 ± 0.03	0.70 ± 0.05

**Table 16:** Pruning-threshold sensitivity.

Threshold	Validation Macro-F1/%	Test Macro-F1/%	Retained Connections
0	92.9	92.2 ± 1.3	55
0.01	93.0	92.4 ± 1.2	42
0.025	93.1	92.5 ± 1.2	32
0.05	92.9	92.4 ± 1.2	24
0.075	92.5	91.8 ± 1.4	18
0.10	91.8	90.7 ± 1.6	12

**Table 17:** Concept-prediction quality on held-out source records.

Concept	Normalized MAE	R^2^	Pearson
Envelope RMS	0.05	0.93	0.96
Envelope kurtosis	0.08	0.87	0.91
BPFI-order energy	0.05	0.94	0.97
BPFO-order energy	0.06	0.92	0.95
BSF-order energy	0.07	0.89	0.93
FTF-order energy	0.08	0.86	0.91
Sideband ratio	0.09	0.85	0.90
Phase concentration	0.06	0.91	0.95
Phase-increment dispersion	0.10	0.84	0.89
Crest factor	0.07	0.89	0.93
Order-spectrum entropy	0.06	0.90	0.94
Mean	0.070	0.891	0.931

## Data Availability

The public datasets analyzed in this study are available from the repositories cited in this manuscript. Restrictions apply to the availability of the field variable-speed acoustic dataset because it contains industrial operating information subject to confidentiality agreements. De-identified event-level metadata, source-record split lists, preprocessing code, model configuration files, and results derived from the public datasets are available from the corresponding author upon reasonable request. Access to de-identified field records may be provided for non-commercial verification purposes with permission from the industrial data owner and subject to an appropriate data-use agreement.

## Abbreviations

The following abbreviations are used in this manuscript:

OSAP-KANFormer	Proposed order-synchronous amplitude–phase diagnostic framework with a concept-based KAN classifier
PC-LAAPR	Physics-constrained learnable analytic amplitude–phase representation
OS-AFEM	Order-synchronous acoustic frame embedding
RAPCA	Reciprocal amplitude–phase cross-attention
KAN	Kolmogorov–Arnold network
MLP	Multilayer perceptron
MHSA	Multi-head self-attention
SD-GLU	Local feed-forward branch using SwiGLU gating and depthwise convolution
LN	Layer normalization
BPFI/BPFO	Ball-pass frequency of the inner/outer race
BSF/FTF	Ball-spin frequency/fundamental train frequency
RMS/MAD	Root mean square/median absolute deviation
$N_{r}$	Number of angular samples per revolution
$T_{\theta }$	Number of angular samples in one diagnostic input
$L_{\theta },R_{\theta }$	Angular-frame length and hop size
${\hat{c}}_{m}$	Predicted value of physical concept m
$f_{km}$	Evidence function from concept m to class k
$r_{km}$	Normalized functional amplitude used for pruning
